# Functional Analysis of the Kinome of the Wheat Scab Fungus *Fusarium graminearum*

**DOI:** 10.1371/journal.ppat.1002460

**Published:** 2011-12-22

**Authors:** Chenfang Wang, Shijie Zhang, Rui Hou, Zhongtao Zhao, Qian Zheng, Qijun Xu, Dawei Zheng, Guanghui Wang, Huiquan Liu, Xuli Gao, Ji-Wen Ma, H. Corby Kistler, Zhensheng Kang, Jin-Rong Xu

**Affiliations:** 1 Purdue-NWAFU Joint Research Center and State Key Laboratory of Crop Stress Biology for Arid Areas, College of Plant Protection, Northwest A&F University, Yangling, Shanxi, China; 2 Department of Botany and Plant Pathology, Purdue University, West Lafayette, Indiana, United States of America; 3 USDA ARS Cereal Disease Laboratory, University of Minnesota, St. Paul, Minnesota, United States of America; University of Melbourne, Australia

## Abstract

As in other eukaryotes, protein kinases play major regulatory roles in filamentous fungi. Although the genomes of many plant pathogenic fungi have been sequenced, systematic characterization of their kinomes has not been reported. The wheat scab fungus *Fusarium graminearum* has 116 protein kinases (PK) genes. Although twenty of them appeared to be essential, we generated deletion mutants for the other 96 PK genes, including 12 orthologs of essential genes in yeast. All of the PK mutants were assayed for changes in 17 phenotypes, including growth, conidiation, pathogenesis, stress responses, and sexual reproduction. Overall, deletion of 64 PK genes resulted in at least one of the phenotypes examined, including three mutants blocked in conidiation and five mutants with increased tolerance to hyperosmotic stress. In total, 42 PK mutants were significantly reduced in virulence or non-pathogenic, including mutants deleted of key components of the cAMP signaling and three MAPK pathways. A number of these PK genes, including Fg03146 and Fg04770 that are unique to filamentous fungi, are dispensable for hyphal growth and likely encode novel fungal virulence factors. Ascospores play a critical role in the initiation of wheat scab. Twenty-six PK mutants were blocked in perithecia formation or aborted in ascosporogenesis. Additional 19 mutants were defective in ascospore release or morphology. Interestingly, *F. graminearum* contains two aurora kinase genes with distinct functions, which has not been reported in fungi. In addition, we used the interlog approach to predict the PK-PK and PK-protein interaction networks of *F. graminearum*. Several predicted interactions were verified with yeast two-hybrid or co-immunoprecipitation assays. To our knowledge, this is the first functional characterization of the kinome in plant pathogenic fungi. Protein kinase genes important for various aspects of growth, developmental, and infection processes in *F. graminearum* were identified in this study.

## Author Summary

Fusarium head blight caused by *Fusarium graminearum* is one of the most important diseases on wheat and barley. Although protein kinases are known to play major regulatory roles in fungi, systematic characterization of fungal kinomes has not been reported in plant pathogens. In this study we generated deletion mutants for 96 protein kinase genes. All of the resulting knockout mutants were assayed for changes in 17 phenotypes, including growth, reproduction, stress responses, and plant infection. Overall, deletion of 64 kinase genes resulted in at least one of the phenotypes examined. In total, 42 kinase mutants were significantly reduced in virulence or non-pathogenic. A number of these protein kinase genes, including two that are unique to filamentous fungi, are dispensable for hyphal growth and likely encode novel fungal virulence factors. Ascospores are the primary inoculum for wheat scab. We identified 26 mutants blocked in ascospore. We also used the *in silico* approach to predict the kinase-kinase interactions and verified some of them by yeast two-hybrid or co-IP assays. Overall, in this study we functionally characterize the kinome of *F. graminearum.* Protein kinase genes that are important for various aspects of growth, developmental, and plant infection processes were identified.

## Introduction

In eukaryotic organisms, reversible protein phosphorylation by protein kinase (PK) is involved in the regulation of various growth and developmental processes and responses to environmental stimuli. Approximately 30% of cellular proteins are phosphorylated [1]. The eukaryotic PK superfamily consists of conventional and atypical protein kinases. Conventional PKs (ePKs) have been classified into eight groups, AGC, CAMK, CK1, CMGC, RGC, STE, TK, and TKL, based on their similarities in amino acid sequences, domain structures, and modes of regulation [2], [3]. Protein kinases with a conserved kinase domain (PF00069) but not classified into these eight groups are categorized as the ‘other’ group of ePKs. Atypical PKs (aPKs) lack significant sequence similarity with ePKs. Four groups of aPKs, Alpha, PIKK, PDHK, and RIO, are known to possess protein kinase activity [2], [3].

In general, approximately 1% of predicted genes encode protein kinases in higher eukaryotes, such as human, mouse, rice, and Arabidopsis [4]–[6]. In the budding yeast *Saccharomyces cerevisiae*, 127 PK genes have been identified, which is approximately 2% of its genome. Many of them play critical roles in signal transduction, cell division, sexual reproduction, and stress responses. The genome of *Schizosaccharomyces pombe* contains 117 PK genes. Approximately 85% of its kinome is shared with *S. cerevisiae*, indicating that these two yeasts have a high degree of homology in their PK genes [7].

To date, genomes of over 40 filamentous fungi have been sequenced. Besides the model filamentous fungi *Neurospora crassa* and *Aspergillus nidulans*, genome sequences are available for a number of plant pathogenic fungi, including *Magnaporthe oryzae*, *Ustilago maydis*, and four *Fusarium* species. In general, less than 1% of the predicted genes in filamentous fungi encode protein kinases [8], [9]. In addition to the well conserved cell-cycle related genes, several PK genes are known by classical genetic studies to be important for hyphal growth in *N. crassa* and *A. nidulans* [10], [11]. In plant pathogenic fungi, a number of PK genes are known to be important for pathogenesis, including the key components of well-conserved MAP kinase (MAPK), calcium, and cAMP signaling pathways [12]–[14]. However, a systematic functional characterization of the kinomes of filamentous fungi or fungal pathogens has not been reported.

Fusarium head blight (FHB) or scab, caused by *Fusarium graminearum,* is one of the most important diseases on wheat and barley [15], [16]. In addition to causing severe yield losses under favorable environmental conditions, this pathogen produces harmful mycotoxins, such as deoxynivalenol (DON) and zearalenone. DON is an important virulence factor in the wheat scab fungus [17], [18]. In addition to its economic importance, *F. graminearum* is a tractable genetic system amenable to molecular and genomic studies. Gene replacement with the split-marker approach is highly efficient [19]. To date, three PK genes, *GPMK1*, *MGV1*, and *SNF1*, have been shown by targeted deletion to be important for various developmental and plant infection processes [20]–[24].

In this study, we identified 116 putative PK genes in *F. graminearum*. Although 20 of them appear to be essential, mutants were generated for the other 96 PK genes and characterized for defects in growth, conidiation, colony and conidium morphology, germination, stress responses, plant infection, DON production, and sexual reproduction. In total, 42 PK mutants were significantly reduced in virulence or non-pathogenic, and 45 mutant were defective in sexual reproduction. A number of these protein kinase genes, including two that are unique to filamentous fungi, are dispensable for hyphal growth and likely encode novel fungal virulence factors. We used the interlog method [25] to predict the PK-PK and PK-protein interaction networks of *F. graminearum*, which has two Cdc2 kinase and two aurora kinase genes. Results from this study indicate that PK genes are important for various developmental and plant infection processes in *F. graminearum*. The functions of some well conserved PK genes, such as *IME2* and *BUB1*, differ significantly between *F. graminearum* and *S. cerevisiae*.

## Results

### Identification of Protein Kinase Genes in *F. graminearum*

**Figure 1:**
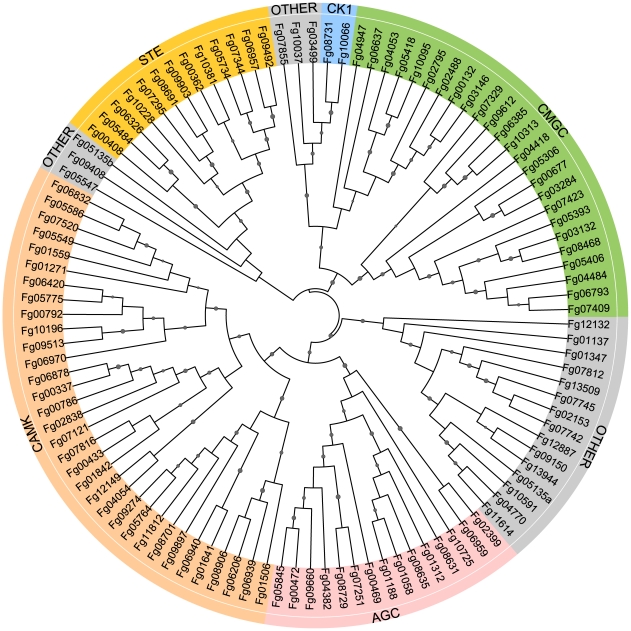
Phylogenetic analysis with the conventional protein kinase (ePK) genes of *Fusarium graminearum.* The unrooted maximum likelihood tree was constructed with the catalytic domain sequences. Different protein kinase groups, AGC (PK**A,** PK**G**, PKC, βARK, ribosomal S6 family PKs and their close relatives), CAMK (calmodulin-regulated kinases), CK1 (casein kinase 1 and close relatives), CMGC (**C**DKs, MAPKs, GSK, and **C**DK-like kinases), STE (many kinases involved in the MAPK cascades), and Other (with a conserved kinase domain but could not be classified) are labeled with different colors. Fg05135 contains two catalytic domains (a and b).

Among the 13,321 predicted genes of *F. graminearum*, 116 encode putative protein kinases (Table S1; Figure 1). Eight of them are atypical PKs. All of the PK genes were manually annotated. Problems with the open reading frame prediction were identified and corrected for 22 of them (Table S1). In comparison with *S. cerevisiae*, *F. graminearum* has fewer PK genes (Table S1). It lacks distinct orthologs of 19 yeast PK genes, including *DBF4*, *SMK1, MEK1, NNK1, ELM1, ALK1, YGK3, NPR1, ISR1,* and *HAL5*. None of them are essential in yeast but some, such as *ALK1* and *ELM1*, are involved in mitosis or cytokinesis. For 22 single copy PK genes in *F. graminearum*, *S. cerevisiae* has two or more paralogs, including *TOR1/TOR2, CKA1/CKA2, DBF2/DBF20, YPK1/YPK2, PKH1/PKH2,* and *RIO1/RIO2* (Table S1). In contrast, *F. graminearum* has two orthologs of *IPL1* (Fg06959 and Fg02399) and *CDC28* (Fg08468 and Fg03132), which are single copy genes in yeast. It also contains 28 putative PK genes, including Fg01058, Fg02488, Fg00792, and Fg01559, that have no distinct orthologs in *S. cerevisiae* (Table S1). Many of them are unique to filamentous fungi.

Interestingly, several PK genes are closely linked in *F. graminearum* (Table S2). For examples, Fg04053 and Fg04054 are only 7626-bp apart. Their chromosomal positions are conserved in *F. verticillioides, F. oxysporum*, and *A. nidulans* but not in *M. oryzae* and *N. crassa*. Fg06939 and Fg06940 encode kinases orthologous to yeast Sat4 (Hal4) and Tos3, respectively. Their orthologs also are closely linked in *F. verticillioides, F. oxysporum*, *M. oryzae*, *A. nidulans*, and *N. crassa* (Table S2).

We searched the PlexDB database (www.plexdb.org) that contains published microarray data of *F. graminearum* [26], [27] to compare expression levels of different PK genes. During barley infection, the expression of 12 PK genes, including Fg06385 (Gpmk1), Fg07295 (Mmk2), Fg07329 (Gsk3), Fg08691 (Pbs2), Fg09660 (Pkc1), and Fg10228 (Swe1) was increased 48 hpi (Table S3). By 144 hpi, their expression levels were up-regulated 5-fold or more. In contrast, no PK genes were reduced over 5-fold during barley infection, although the expression of Fg01559, Fg07745, Fg09150, and Fg07344 was reduced approximately 2-fold at 144 hpi (Table S3).

During spore germination, Fg03132 is highly expressed at early stages. In comparison with ungerminated conidia, it was up-regulated over 11- and 8-fold at 2 and 8 h, respectively. The other *CDC28* ortholog, Fg08468, also was up-regulated but to a lesser extent. Its expression was increased 2.9- and 2.0-fold at 2 and 8 h, respectively (Table S3). The expression of Fg06502 (Rio1), Fg01347 (Bub1), and Fg00472 (Sch9) also peaked at 2 h. These genes may play a role in the establishment of polarized growth. Fg01271 (Cdc5), Fg13318 (Mec1), Fg09408 (Kin3), and Fg08635 (Dbf2) were up-regulated at 2 and 8 h, when cell division and cytokinesis are activated during germination.

### Knockout Mutants of Predicted PK Genes

The *gpmk1* and *mgv1* mutants were generated in previous studies [20], [22], [24]. For all of the other PK genes, gene replacement constructs were generated by the split-marker approach [19] and transformed into protoplasts of the wild-type strain PH-1. The resulting hygromycin-resistant transformants were screened by PCR with primers F5 and R6 (Figure S1) located in the deleted region. All putative knockout mutants were further confirmed by PCR with primer pairs F7/H855R and R8/H856F [28]. Primers F7 and R8 were located outside the flanking sequences of the gene replacement constructs (Figure S1). Only transformants that underwent homologous recombination in the flanking sequences contain PCR products of expected sizes. A total of 20 PK knockout mutants (Table S1) were selected for verification by Southern blot hybridizations. All of them, including the Fg00362, Fg04053, Fg08701, Fg08906, Fg10228, and Fg10381 mutants (Table S1), were confirmed to be true deletion mutants.

For 96 PK genes, we were able to identify at least two or more knockout mutants with similar phenotypes described below. Twelve of them are orthologous to essential genes in *S. cerevisiae* or *S. pombe* (Table 1), indicating that these protein kinases are not required for hyphal growth in *F. graminearum*. However, many of these mutants grew poorly. Of the 20 PK genes for which we failed to identify knockout mutants, at least 35 transformants from three or more independent transformations were screened (Table 1), indicating that knockout mutants may be nonviable. Sixteen of them are orthologous to essential genes in *S. cerevisiae*, including *FgPKC1* (Fg09660), *FgTRA1* (Fg06089), *FgKIN28* (Fg07423), and *FgHRR25* (Fg08731). For the Fg05306, Fg05775, Fg06637, and Fg05393 genes, their orthologs in yeast are not essential but we failed to identify knockout mutants after screening over 60 transformants from at least three independent transformations (Table 1). Deletion of these genes may be lethal because of the gene replacement efficiency of the split-marker approach in *F. graminearum*.

**Table 1:** Protein kinase genes essential in *S. cerevisiae*, *S. pombe,* or *F. graminearum*.

*F. graminearum*	*S. cerevisiae*	*S. pombe*	Transformants screened	Mutants identified
**PK genes essential in the fission or budding yeast but not in ** ***F. graminearum***				
Fg02399 a	*IPL1*	*ark1*	20	14
Fg00433	*RAD53*	*cds1*	20	11
Fg04947	*CAK1*	-	16	6
Fg13318	*MEC1*	SPBC216.05	20	10
Fg10381	*CDC15*	*cdc7*	20	3
Fg08468 a	*CDC28*	*cdc2*	18	12
Fg03132 a	*CDC28*	*cdc2*	20	8
Fg01188	*CBK1*	*orb6* c	87	7
Fg05734	*KIC1*	*nak1* c	20	5
Fg07344	-	*sid1*	20	15
Fg08906	-	SPCC70.05C	20	10
Fg04053	-	*prp4*	70	7
**PK genes essential in ** ***F. graminearum*** ** and other fungi**				
Fg00677	*CKA1, CKA2*	*cka1*	75	0
Fg01137	*MPS1*	*mph1*	40	0
Fg01271	*CDC5*	*plo1*	36	0
Fg04054	*VHS1*, *SKS1*	*ran1*	85	0
Fg05845	*YPK1, YPK2*	*gad8*	52	0
Fg06089	*TEL1*	*tel1*	105	0
Fg06502	*RIO1, RIO2*	SPAC10F6.10,SPBC1703.05	46	0
Fg06959 a	*IPL1*	*ark1*	37	0
Fg07409	*SGV1*	*cdk9*	64	0
Fg07423	*KIN28*	*crk1, mcs6*	38	0
Fg07855	*CDC7*	*hsk1, spo4*	50	0
Fg08133	*TOR1, TOR2*	*tor1, tor2*	100	0
Fg08731	*HRR25*	*hhp1, hhp2*	37	0
Fg09408 (*nimA*)^b^	*KIN3*	*fin1*	36	0
Fg09660	*PKC1*	*pck1, pck2*	40	0
Fg10725	*PKH1,PKH2*	*ksg1*	42	0
Fg05393	*PHO85* d	*pef1*	141	0
Fg06637	*KNS1* d	*lkh1*	95	0
Fg05775	*IRE1* d	*ppk4*	117	0
Fg05306	*VPS15* d	*ppk19*	64	0

a One of the two paralogs in *F. graminearum*. -, no distinct ortholog.

b Orthologous to *nimA,* an essential gene in *A. nidulans*.

c In *S. pombe*, *orb6*, *nak1*, and *sid1* are essential genes. *KIC1* and *CBK1* are essential genes in the wild-type but no *ssd1* mutant strains of *S. cerevisiae*. Fg07344 and Fg05734 are orthologous to Sid1 and Nak1 of *S. pombe*, respectively. Sid1 has no distinct ortholog in *S. cerevisiae*.

d *PHO85, KNS1, IRE1*, and *VPS15* are not essential genes in *S. cerevisiae*.

All of the knockout mutants were characterized for defects in vegetative growth, colony morphology, pigmentation, conidiation, conidium morphology, conidium germination and germ tube growth, hyphal tip growth and branching, perithecium formation, ascospore production, ascospore dispersal, DON production, wheat and corn infection, and responses to treatments with 0.05% H_2_O_2_, and 0.7 M NaCl. The resulting phenotypic data were deposited in a searchable database available at fgkinome.nwsuaf.edu.cn. Overall, deletion of 64 non-essential PK genes (66.7%) resulted in at least one of the 17 phenotypes examined. Because of the importance of protein kinases in fungal growth and differentiation, many of these mutants have pleiotropic defects, and we were able to isolate at least one PK mutant defective in each phenotype examined in this study. When analyzed for the association between different phenotypes by Pearson correlation efficient, defects in plant infection and vegetative growth had the highest correlation efficient followed by the correlation between sexual reproduction and growth rate or virulence (Table S4). Although phenotypes of at least two knockout mutants were examined for each gene, we have selected nine genes (Fg04053, Fg05734, Fg07251, Fg02795, Fg07329, Fg07344, Fg08906, and Fg10381) for complementation assays. For all of them, the reintroduction of the wild-type allele rescued the defects observed in the corresponding mutants.

### PK Genes Important for Hyphal Growth

**Figure 2:**
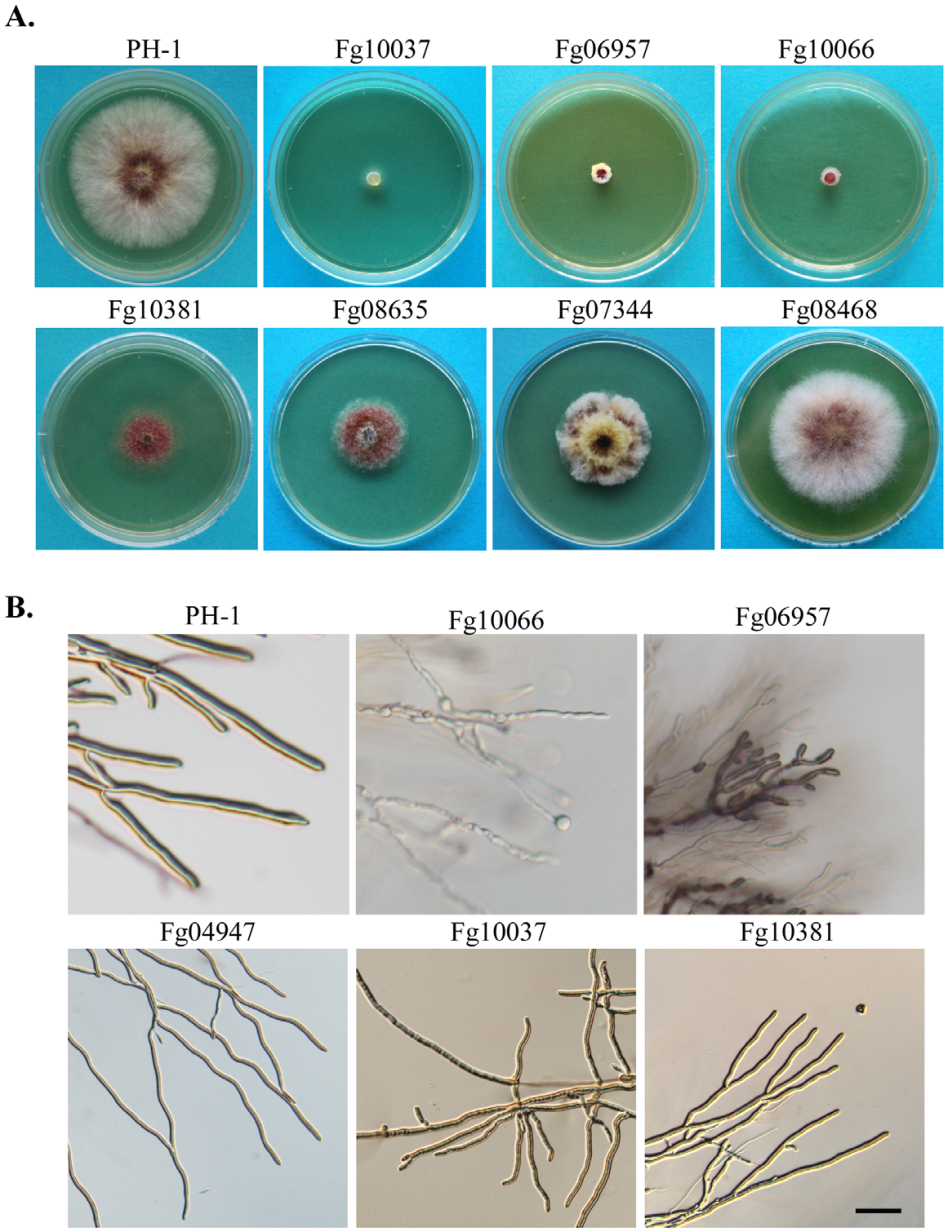
Representative PK mutants with defects in colony morphology and hyphal growth. **A.** Three-day-old PDA cultures of PH-1 and the Fg10037, Fg06957, Fg10066, Fg10381, Fg08635, Fg07344, and Fg08468. **B.** Hyphae of PH-1 and the Fg10066, Fg06957, Fg04947, Fg10037, and Fg10381 mutants grown on CM slab agars for 36 h. Bar = 100 µm.

Among the 96 non-essential PK genes, 32 of them (66.7%) were found to play critical roles in vegetative growth. Deletion of any one of these genes, including *GzSNF1* and *MGV1* [20], [29], resulted in over 30% reduction in growth rate (Table 2). Many of these mutants had abnormal colony morphology, growth, or branching patterns (Figure 2A; fgkinome.nwsuaf.edu.cn). The Fg00362, Fg01188 (Cbk1), and Fg04053 mutants had the most significant reduction in growth (>90%) and formed compact colonies with limited hyphal growth (Figure S2). The Fg00362 and Fg01188 mutants had similar morphological defects and produced densely aggregated vegetative hyphae that were wider and had shorter compartments and fewer branches. Their orthologs in *N. crassa* are the *POD-6* (polarity-defective 6) and *COT-1* genes that are functionally related in regulating hyphal growth [10]. The Fg04053 mutant appeared to have less severe defects in hyphal morphology and branching (Figure S2). It formed non-pigmented colonies with rare aerial hyphae. Like Fg00362, Fg04053 lacks a distinct ortholog in *S. cerevisiae*.

**Table 2:** Protein kinase genes important for vegetative growth and/or pathogenesis.

Strains^a^	Growth rate (%)^b^	Conidiation (%)^b^	Disease Index^c^
PH-1 (WT)	100.0	100.0	13.6
Fg04484 (Srb10)	69.4	59.9	1.0
Fg05547 (Atg1)	67.8	45.9	2.0
Fg04382 (Kin82)	67.4	69.4	7.5
Fg08468 (Cdc28)	66.7	131.6	1.5
Fg09903 (Ste7)	65.9	151.0	1.0
Fg06939 (Sat4)	64.4	52.3	4.5
Fg05734 (Kic1)	63.9	38.5	1.8
Fg13318 (Mec1)	62.4	65.0	1.2
Fg06878 (Cmk1/Cmk2)	60.4	73.9	2.1
Fg08701 (Gin4-like)^c^	58.1	50.0	8.5
Fg10228 (Swe1)	55.9	27.1	1.3
Fg10381 (Cdc15)	52.8	17.8	0.9
Fg08635 (Dbf2/Dbf20)	50.4	14.	0.8
Fg01641 (Sak1)	49.4	10.0	1.2
Fg06385 (Gpmk1)	47.0	32.6	0.5
Fg08691 (Pbs2)	64.41	41.2	0.8
Fg05484 (Ste11)	45.6	73.6	1.0
Fg04947 (Cak1)	39.0	22.4	1.7
Fg02795 (Sky1)	38.9	62.2	0.5
Fg07295 (Mkk1/Mkk2)	30.0	50.9	0.7
Fg06326 (Bck1)	29.3	21.5	0.0
Fg10313 (Mgv1)	28.2	69.7	0.0
Fg09897 (Snf1)	25.3	55.9	1.0
Fg00408 (Ssk2/Ssk22)	42.4	54.5	0.5
Fg07251 (Tpk2)	17.1	47.9	1.0
Fg07329 (Gsk3)	15.7	2.7	0.5
Fg10037 (Bud32)	15.3	1.4	0.0
Fg06957 (Cla4)	15.2	53.0	2.1
Fg10066 (Yck1/2/3)	14.8	0.0	0.9
Fg01188 (Cbk1)	4.4	0.0	0.1
Fg00362	2.8	0.0	0.0
Fg04053	2.0	1.3	0.0
Fg09274 (Kin1/Kin2)	80.7	29.6	3.5
Fg00472 (Sch9)	86.1	58.9	2.6
Fg04770	94.7	111.8	2.5
Fg10095	78.8	70.0	2.3
Fg06793 (Ctk1)	74.5	52.9	2.0
Fg01312 (Rim15)	103.7	3.2	2.0
Fg09612 (Hog1)	44.9	90.6	1.5
Fg03284 (Cka1/Cka2)	89.3	105.9	1.5
Fg05418 (Yak1)	101.2	30.5	1.3
Fg08906 (Prr2)	103.9	62.5	1.4
Fg07344	74.0	16.8	1.0
Fg11812 (Kin4)	80.9	52.0	1.0

a Mutants deleted of the predicted PK genes. Yeast orthologs, if exist, were listed in the bracket.

b Percentage of growth rate and conidiation of the mutants in comparison with that of PH-1.

c Diseased spikelets per wheat head examined 14 dpi. Only mutants with >30% reduction in growth rate or a disease index <5 were listed.

Several protein kinase genes, including *FgCAK1* (Fg04947) and Fg10066, were found to be important for normal hyphal morphology. The *Fgcak1* deletion mutant produced wavy hyphae with reduced branching (Figure 2B). In the Fg10066 mutant, hyphae became narrower and often had swollen tips (Figure 2B). Fg10066 is the only ortholog of three yeast paralogous CK1 (casein kinase 1) genes *YCK1, YCK2,* and *YCK3*. In *S. cerevisiae*, the *yck1 yck2 yck3* triple deletion mutant is nonviable. *F. graminearum* has only two CK1 genes, Fg10066 and Fg08731. Fg08731, like its yeast ortholog *HRR25*, is an essential gene in *F. graminearum* (Table 1).

The *Fgbud32* (Fg10037) deletion mutant was reduced in aerial hyphal growth and produced whitish colonies (Figure 2). In yeast, *BUD32* regulates bud site selection. Although deletion of *BUD32* is not lethal in *S. cerevisiae*, its ortholog is an essential gene in *S. pombe*. In *F. graminearum*, hyphal branching was reduced in the *Fgbud32* mutant. However, it often had bifurcated hyphal tips and displayed clustered branching (Figure 2), suggesting that *FgBUD32* plays an important role in hyphal branching. The *Fgcdc15* (Fg10381) and *Fgsky1* (Fg02795) mutants also were reduced in hyphal branching and produced less aerial hyphae that the wild type, but they grew faster than the *Fgbud32* mutant (Figure 2).

### Protein Kinases Involved in Asexual Reproduction

**Figure 3:**
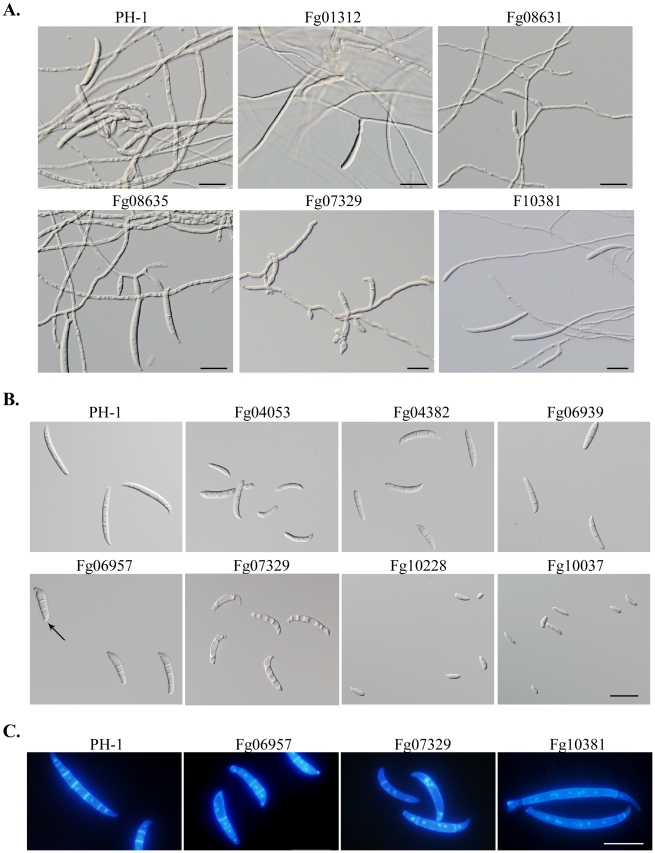
Mutants with defects in conidiation and conidium morphology. **A**. Five-day-old CMC cultures of PH-1 and the Fg01312, Fg08731, Fg08635, Fg07344, and Fg10381 mutants with defects in conidiogenesis. Arrows point to phialides and conidiophores. **B**. Conidia of PH-1 and the Fg04382, Fg06939, Fg04053, Fg10228, Fg06957, and Fg07329 mutants with defects in conidium morphology. Arrows point to foot cells. **C.** Conidia of PH-1 and the Fg06957, Fg07329, and Fg10381 mutants were stained with DAPI and Calcofluor. Bar = 20 µm.

In *F. graminearum*, conidia are formed either directly on short hyphal branches or on phialides that are often formed in clusters in liquid cultures. Three PK genes, Fg00362, Fg01188, and Fg10066, are found to be essential for conidiation. In addition, 33 PK mutants were reduced in conidiation by over 50% in comparison with PH-1 (Table 2). Conidia were rarely formed by the Fg01312, Fg04053, Fg007329, and Fg10037 mutants (Table 2). For most of these conidiation mutants, their growth rate also was significantly reduced. In fact, among the mutants with over 80% reduction in growth rate, only the *Fgtpk2* (Fg07251) mutant was reduced less than 80% in conidiation. However, the *Fgrim15* (Fg01312) and Fg08631 (Ypk2-like) deletion mutants were reduced over 90% in conidiation but had no obvious defects in vegetative growth. These two PK genes may be important for conidiophore development or conidiogenesis. The *Fgrim15* and Fg08631 mutants often formed conidia directly on short hyphal branches (Figure 3A). Clusters of phialides were rarely observed, which may be responsible for reduced conidiation. The *Fgcdc15* (Fg10381) mutant also was significantly reduced in conidiation. It often formed conidia directly at the hyphal tips (Figure 3A), indicating that septation is important for conidiophore or phialide development.

The *Fgswe1*, *Fgbud32*, Fg06939, Fg04053, *Fgfpk1* (Fg04382), *Fggsk3* (Fg07329), and *Fgcla4* (Fg06957) mutants formed smaller or shorter conidia with abnormal morphology (Figure 3B). All the conidium morphology mutants also were significantly reduced in conidiation, indicating that these six PK genes may be involved in the regulation of conidium development and maturation processes. Whereas conidia of the Fg06939, *Fgpfk1,* and Fg04053 mutants tended to have four compartments, most of conidia produced by the *Fgswe1* mutant were single- or two- celled (Figure 3B), similar to microconidia produced by other Fusarium species. Conidia of the *Fggsk3* mutant were highly vacuolated and had curved apical compartments and less septation (Figure 3C). Interestingly, although the *Fgcdc15* mutant produced conidia with normal size and morphology, it also had less septation in conidia (Figure 3C). It often had only one or two septa towards the ends of conidia.

### Conidium Germination and Germ Tube Growth

Among 93 PK mutants that produced conidia, none of them was blocked in conidium germination. Interestingly, in the Fg04053 mutant, approximately 5% of freshly harvested conidia had germinated in sporulating cultures. This PK gene may play a role in self-inhibition of conidium germination in the spore-producing cultures. After germinating for 12 h, many PK mutants with growth defects produced shorter germ tubes than the wild type. Nine of them, including the Fg10228, Fg00479, Fg00472, Fg09897, Fg10228, Fg07251, and Fg07329 mutants, had the most significant defects in germ tube morphology, growth, or branching (Table S5). In the Fg01641 and Fg09897 mutants, some conidium compartments produced more than one germ tube, resulting in an increase in the number of germ tubes produced by individual conidia.

### Sexual Reproduction and Ascospore Discharge

*F. graminearum* is a homothallic fungus and ascospores play a critical role in its infection cycle as the primary inoculum. When assayed for sexual reproduction on carrot agar plates, most of the PK mutants (51) were normal in the production of perithecia and cirrhi. A total of 20 PK mutants failed to produce perithecia (Table 3). Six of them were mutants in genes of the Mgv1 and Gpmk1 MAPK pathways, which are known to be required for sexual reproduction in *F. graminearum* [20], [22], [24]. Interestingly, mutants deleted of the Fg00408, Fg08691, and Fg09612 genes that are orthologous to yeast *SSK22*, *PBS2*, and *HOG1* [30] were also blocked in perithecium formation (Table 3). These results indicate that all three MAPK pathways are important for sexual reproduction in *F. graminearum*.

**Table 3:** Phenotypes of the 96 protein kinase mutants in sexual reproduction.

Defects in Sexual Reproduction		Mutants deleted of *F. graminearum* genes
**No defects** (same as the wild-type strain PH-1) (51 genes)		Fg00132, Fg12149, Fg09513, Fg03499, Fg12887, Fg00469, Fg07520, Fg11614, Fg04418, Fg03284, Fg04416, Fg09150, Fg01559, Fg02838, Fg05135, Fg12132, Fg05406, Fg02488, Fg07742, Fg06940, Fg07121, Fg08729, Fg02153, Fg00472, Fg07812, Fg04770, Fg10591, Fg09492, Fg05549, Fg02399, Fg00433, Fg05764, Fg07816, Fg06206, Fg00786, Fg03132, Fg13944, Fg01963, Fg06939, Fg03146, Fg13509, Fg06420, Fg06832, Fg10196, Fg00792, Fg08631, Fg07381, Fg07745, Fg01312, Fg05586, Fg05519
**No perithecium formation**		Fg06326, Fg07295, Fg10313, Fg05484, Fg09903, Fg06385, Fg00408, Fg08691, Fg06970, Fg02795, Fg04484, Fg05547, Fg10066, Fg01188, Fg10037, Fg07329 , Fg00362, Fg10381, Fg09612, Fg04053
**Formed perithecia but no cirrhi**		
**Type I**: defective in ascospore formation	Aborted in ascus development	Fg10228, Fg08635
	No ascogenous tissues observed a	Fg04947, Fg05734, Fg06793, Fg08701
**Type II**: defective in ascospore release	Normal ascospores	Fg01506, Fg13318, Fg08906, Fg01842, Fg06957, Fg10095
	Defective in ascospore morphology or development b	Fg01347, Fg04382, Fg09274, Fg01641, Fg07251, Fg01058, Fg07344, Fg08468, Fg09897, Fg11812 (Kin4), Fg05418, Fg06878, Fg00337.

a These mutants tended to produce smaller and fewer perithecia.

b The Fg01641, Fg07251, Fg07344, and Fg01058 mutants were defective in ascospore morphology. In the Fg09274 mutant, ascospores germinated and produced long germ tubes that tangled together inside perithecia. All the other mutants were reduced in ascosporogenesis. Normal ascospores were rarely observed in the Fg08468, Fg01347, and Fg04382 mutants.

**Figure 4:**
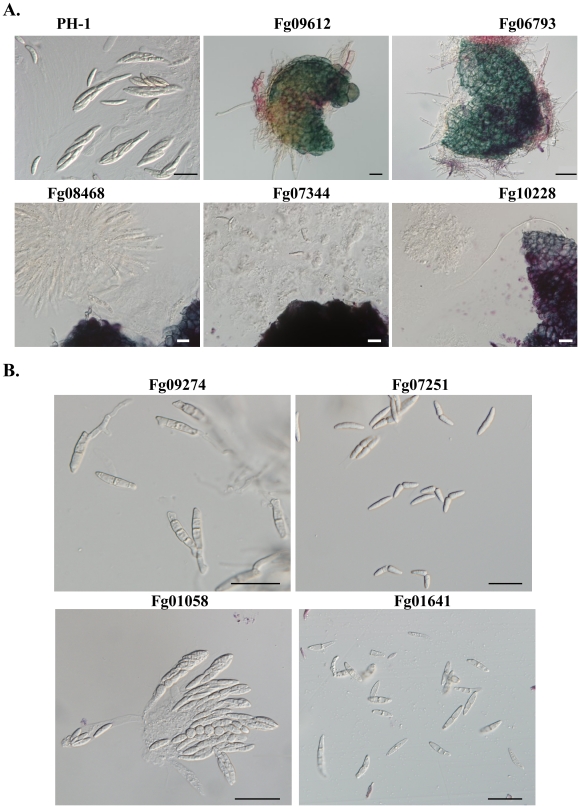
Protein kinase mutants with defects in sexual reproduction. All mating cultures were incubated at 25°C under black light. **A.** Asci and ascospores were not formed in perithecia produced by the Fg09612, Fg06793, Fg08468, Fg07344, and Fg10228 mutants. **B**. Ascospores of the Fg09274, Fg07251, Fg01058, and Fg01641 mutants. Bar = 20 µm.

The 26 PK mutants that formed perithecia but failed to produce cirrhi could be divided into two types. Type I mutants were defective in the development of ascogenous hyphae, asci, or ascospores even after prolonged incubation (Figure 4A; Table 3). The Fg04947, Fg05734, Fg06793, and Fg08701 mutants produced a few small perithecia that were blocked in the development of ascogenous hyphae. In contrast, the *Fgdbf2* (Fg08635) and *Fgswe1* (Fg10228) mutants produced morphologically normal perithecia that contained aborted ascogenous tissues (Figure 4A). Type II mutants were blocked in ascospore release. These mutants formed ascospores inside perithecia (Figure 4A) but failed to produce cirrhi after incubation for one month or longer. Among them, the Fg08468, Fg07344, Fg06878 (Cmk1/2) and Fg10095 mutants were significantly reduced in ascospore formation. They produced only a few ascospores per perithecium. In the Fg08468 mutant, fascicles of aborted asci with no mature ascospores were observed, indicating that Fg08468 is important for ascosporogenesis. In addition, we found that ascospores formed by the Fg07251 (Tpk2), Fg01641 (Sak1), and Fg01058 (Cbk1-like) mutants had morphology defects. While ascospores of the Fg07251 mutant were often fragmented in the middle, the Fg01641 mutant produced highly vacuolated ascospores (Figure 4B). For the Fg01058 mutant, some ascospores appeared be single-celled and spherical (Figure 4B). Normal mature ascospores are four-celled in *F. graminearum*. Interestingly, most of the ascospores formed by the Fgkin1 (Fg09274) mutant had germinated or were germinating inside perithecia (Figure 4B). *FgKIN1* and vesicle trafficking must play a critical role in preventing ascospores from germination before being released.

### Mutants with Altered Responses to Hyperosmotic Stress

**Figure 5:**
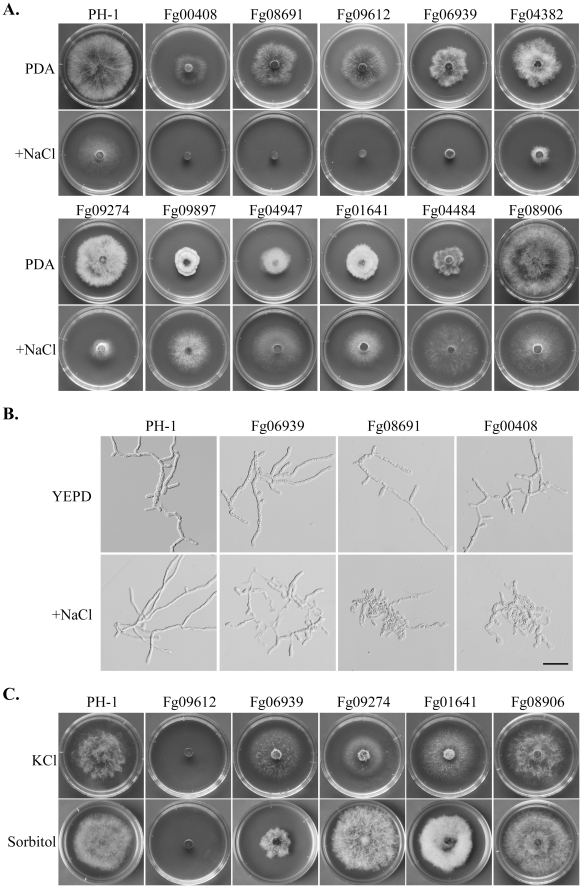
Mutants with altered sensitivity to hyperosmotic stress. **A**. Colonies of PH-1 and the Fg00408, Fg08691, Fg09612, Fg06939, Fg04382, Fg09274m Fg09897, Fg04947, Fg01641, Fg04484, Fg08906 mutants formed on CM with (upper row) or without (lower row) 0.7 M NaCl after incubation for 3 days. **B**. Defects in hyphal growth of the Fg06939, Fg08691, and Fg00408 mutants in the presence of 0.7 M NaCl. **C.** Cultures of PH-1, Fg09612, Fg06939, Fg09274, Fg01641, and Fg08906 grown on CM with 0.7 M KCl or 1 M sorbitol.

The Fg00408, Fg08691, and Fg09612 mutants had no obvious growth after incubation for 3 days on CM with 0.7 M NaCl (Figure 5A). When examined microscopically, germ tubes of these mutants were significantly stunted with NaCl treatment (Figure 5B). These mutants also were hypersensitive to 1 M sorbitol and 0.7 KCl (Figure 5C), indicating that the MAPK cascade orthologous to the yeast Hog1 pathway is conserved in *F. graminearum* for regulating responses to hyperosmotic stress. Like osmoregulation MAPK pathway mutants, the *Fgfpk1* (Fg04382) mutant was hypersensitive to 0.7 M NaCl (Figure 5A), indicating that proper regulation of phospholipid translocation also plays a role in normal response to hyperosmotic stress.

The *Fgsat4* (Fg06939) and *Fgkin1* (Fg09274) mutants also had increased sensitivity to 0.7 M NaCl (Figure 5A) but they were normal in response to 1 M sorbitol (Figure 5C). However, the *Fgsat4* mutant was more tolerant to 0.7 M KCl than the wild type (Figure 5C). In yeast, Sat4 kinase is involved in salt tolerance by regulating the Trk1-Trk2 potassium transporters [31]. *FgSAT4* may be specifically involved in the regulation of K^+^/Na^+^ transporter genes in *F. graminearum.* In contrast, the *Fgkin1* mutant was hypersensitive to 0.7 M KCl. The presence of 0.7 M KCl but not 1 M sorbitol inhibits its growth (Figure 5C). In *S. pombe*, the *kin1* mutant expresses increased sensitivity to excess chloride ion [32]. These results indicate that *FgSAT4* and *FgKIN1* are not directly involved in osmoregulation, but they play key roles in avoiding K^+^ and Cl^-^ toxicity in *F. graminearum*.

Interestingly, several PK gene deletion mutants had increased tolerance to hyperosmotic stress (Figure 5A; Table S6). Two of them, the *Fgsrb10* (Fg04484) and *Fgprr2* (Fg08906) mutants, grew faster than PH-1 under hyperosmotic conditions. The *Fgprr2* mutant was normal in growth, but the *Fgsrb10*, *Fgcak1* (Fg04947), and *Fgsnf1* (Fg09897) mutants were reduced in growth rate on regular medium, but addition of 0.7 M NaCl suppressed their growth defects. In *S. cerevisiae*, *SNF1* is required for the expression of glucose-repressed genes, thermotolerance, and peroxisome biogenesis. Like its orthologs in other plant pathogenic fungi, *GzSNF1* is important for vegetative growth, sexual reproduction, and pathogenesis [29]. However, it is not known whether *SNF1* orthologs are involved in tolerance to hyperosmotic stress in fungi. Fg01641 is orthologous to yeast Sak1, which is an upstream kinase for the Snf1 complex. In yeast, the Cak1 kinase is responsible for the activation of Srb10, which is a kinase converges with Snf1 on the Sip4 transcriptional activator [33]. Therefore, it is possible that some of these genes are functionally related in *F. graminearum* to negatively regulate subsets of genes involved in response to hyperosmotic stress.

### PK Genes Involved in Response to Oxidative Stress

**Figure 6:**
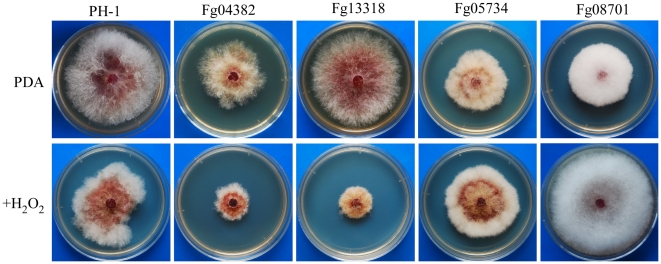
Mutants with defects in response to oxidative stress. Colonies formed by the wild type (PH-1) and Fg04382, Fg13318, Fg05734, and Fg08701 mutants on PDA with (upper) or without (lower) 0.05% H_2_O_2_ after incubation for 5 days.

In comparison with the wild type, the Fg00472, Fg04382, Fg05418 (Yak1), and Fg13318 mutants were hypersensitive to oxidative stress (Table S6). Their growth was more severely reduced by 0.05% H_2_O_2_ than that of the wild type (Figure 6). Although to a less extent, the *Fgssk2*, *Fgpbs2*, and *Fghog1* mutants also were more sensitive to H_2_O_2_ than the wild type (Table S6), indicating that the osmoregulation pathway is also involved in regulating responses to oxidative stress. The *Fggsk3* (Fg07329) and *Fgbud32* (Fg10037) mutants had no visible hyphal growth in the presence of 0.05% H_2_O_2_. However, growth of these two mutants was severely reduced on regular PDA. Interestingly, H_2_O_2_ treatment inhibited conidium germination in the *Fggsk3* but not the *Fgbud32* mutant. After incubation for 24 h with as low as 0.01% H_2_O_2_, conidium germination was not observed in the *Fggsk3* mutant. Therefore, *FgGSK3* may play a role in response to oxidative stress during conidium germination.

In contrast, the *Fgkic1* (Fg05734) and Fg08701 mutants were more tolerant to oxidative stress than the wild type (Table S6). The Fg08701 mutant became almost insensitive to hydrogen peroxide_._ In the presence of 0.05% H_2_O_2_, it grew faster than the wild type (Figure 6). Fg08701 encodes a Gin4-like kinase but it has no distinct ortholog in the fission or budding yeast. Deletion of Fg08701 may result in enhanced expression of genes involved in ROS scavenging.

### Plant Infection and Virulence

**Figure 7:**
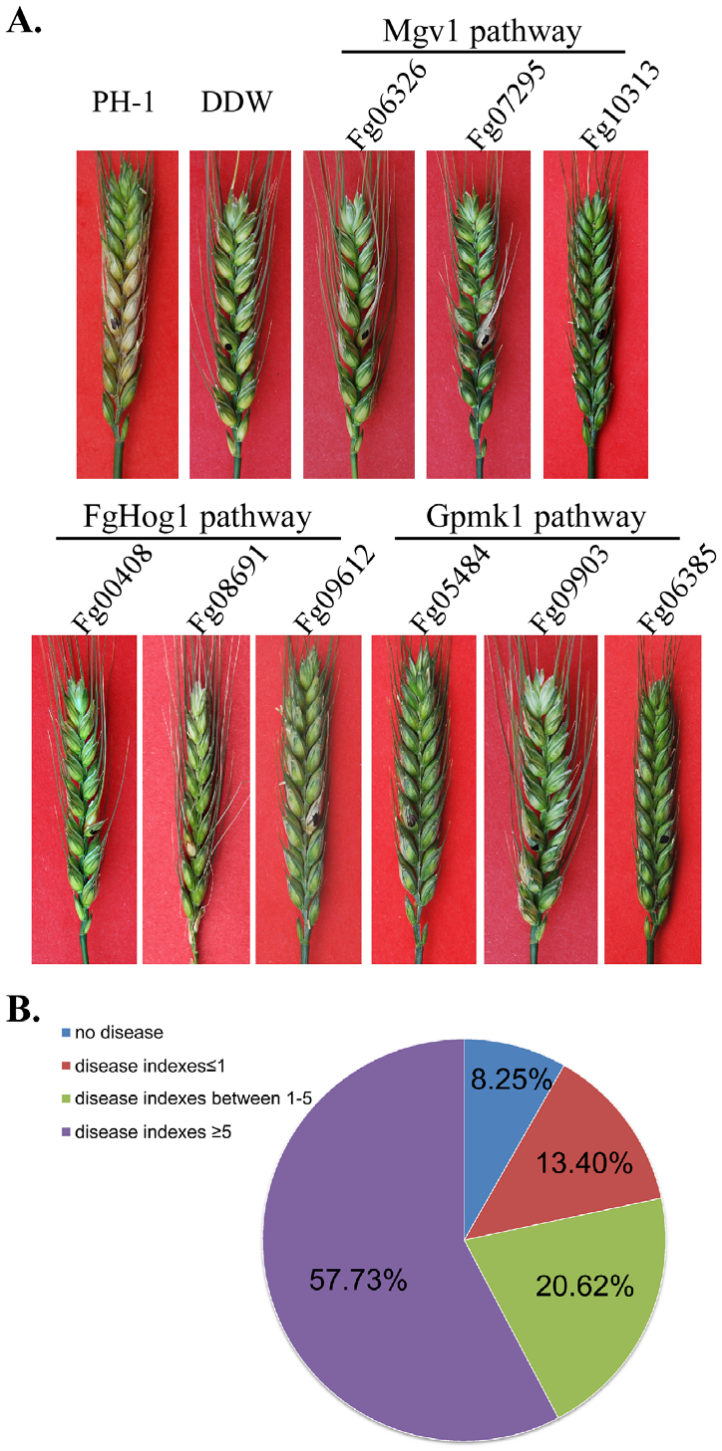
Infection assays with flowering wheat heads. **A**. Typical wheat heads infected with the wild-type strain PH-1 and mutants blocked in the three MAPK pathways. **B**. Categories of mutants with different disease indices.

In infection assays with flowering wheat heads, 42 PK deletion mutants were found to have a disease index less than 5 (Table 2; Figure 7A). Under the same conditions, the wild type had a disease index of approximately 14. Among them, 22 PK mutants were found to be non-pathogenic or caused symptoms only on the inoculated kernels, indicating defects in colonization or spreading. In *F. graminearum*, the Gpmk1 and Mgv1 MAPK genes are known to be important for plant infection. Thus, it is not surprising that other components of these two MAPK pathways are required for plant infection (Figure 7B). Interestingly, the *Fgssk22*, *Fgpbs2*, and *Fghog1* mutants also were defective in plant infection, indicating that the osmoregulation pathway may play a critical role in overcoming plant defense responses and infectious growth in *F. graminearum*.

Like many other filamentous ascomycetes, *F. graminearum* has two genes encoding the catalytic subunits of protein kinase A (Fg07251 and Fg08729). Fg07251 is orthologous to CpkA of *M. oryzae* and its orthologs in other fungal pathogens that are known to be essential for plant infection [13], [34]. The Fg07251 mutant was non-pathogenic but it, unlike the *cpkA* mutant, was significantly reduced in growth. In contrast, the Fg08729 mutant had no detectable phenotype, suggesting that it plays a minor role in PKA activities.

Besides genes related to the signaling pathways, 16 PK genes, including Fg10381, Fg10066, Fg07344, Fg00362, Fg04053, Fg01188, Fg02795, Fg07329, Fg08635, Fg04484, Fg09897, Fg11812, Fg10037, Fg01641, Fg13318, and Fg05418, were essential for spreading from inoculated kernels to nearby spikelets. They had a disease index less than 1.5. Many of them, such as the Fg00362 and Fg01188 deletion mutants, were significantly reduced in growth rate, which may contribute to their reduced virulence. When analyzed for the association between reduced virulence and other phenotypes, it is not surprising that defects in plant infection and vegetative growth were found to have the highest correlation efficient (Table S4). Among all of the 32 mutants with over 30% reduction in growth rate, only the *Fgkin82* (Fg04382) and Fg08701mutants had a disease index greater than 7 (Table 2). These two genes may have different functions during vegetative growth and infectious growth. However, among the mutants with a disease index less than 5, four PK mutants (Fg05418, Fg01312, Fg04770, and Fg08906) were not significantly affected vegetative growth. In addition, the Fg09274, Fg07344, Fg00472, Fg10095, Fg06793, Fg03284, and Fg11812 deletion mutants were reduced less than 30% in growth rate (Table 2), indicating that defects other than growth rate may be responsible for reduced virulence in these mutants.

### DON Production

For mutants with a disease index larger than 1.5, infected wheat kernels were harvested and assayed for DON production. Except for the Fg04947 mutant that produced barely detectable amounts of DON, all other mutants assayed produced significant amounts of DON (>400 ppm) in infested kernels (Table S7). However, DON production was reduced in many of these PK mutants. In 22 mutants, the level of DON in infested wheat kernels was less than 900 ppm. Among them, eight mutants had a disease index less than 5 (Table S7). These results indicate that reduction in DON production was positively correlated with changes in virulence in most of these PK mutants, which is consistent with the importance of DON in plant infection [17], [18]. However, a few PK mutants had no significant changes in DON production, such as the Fg06957 and Fg05547 mutants, but were drastically reduced in virulence (Table S7). Factors other than DON production, such as defects in growth and stress responses, may be responsible for reduced virulence in these mutants.

### Predicted Networks of PK-PK and PK-Protein Interactions

**Figure 8:**
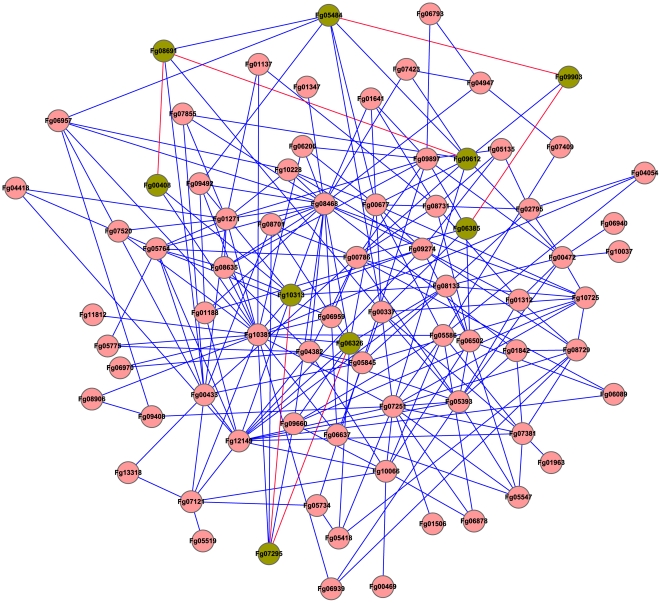
The predicted interactome of protein kinases in *F. graminearum*. Orthologs of yeast protein kinases (PKs) were identified and used for the prediction of protein-protein interactions. The PK-PK interaction map was generated with Cytoscape. Three MAP kinase pathways are highlighted in green.

**Figure 9:**
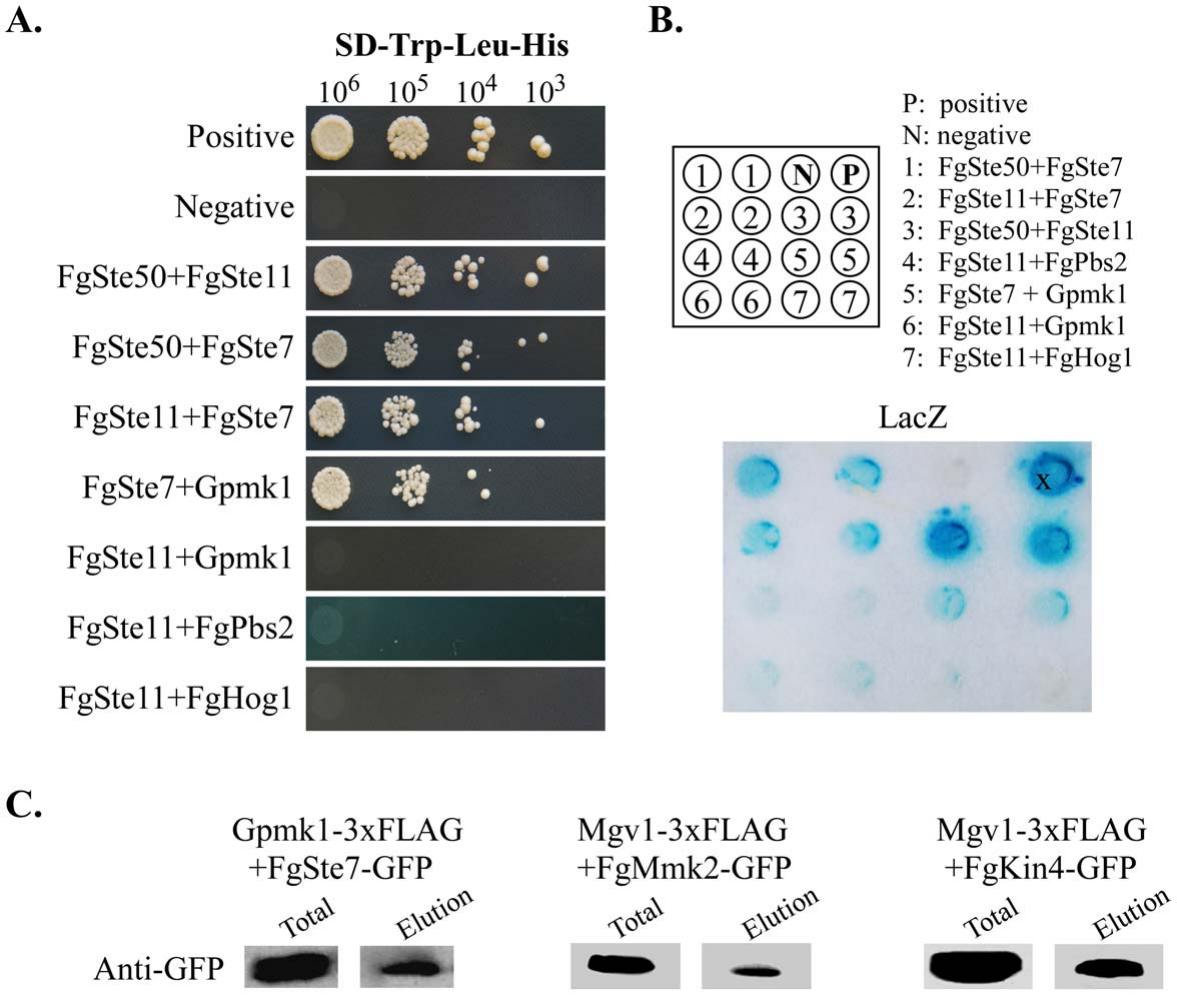
Verification of predicted PK-PK interactions. **A**. Different concentrations of yeast cells (cells/ml) of the transformants expressing the labeled bait and prey constructs were assayed for growth on SD-Leu-Trp-His plates. P and N were the positive and negative controls provided in the BD Matchmaker library construct kit. **B**. The same set of yeast transformants were assayed for β-galactosidase activities. **C.** Co-IP assays. Western blots of total proteins isolated from transformants expressing the GFP and 3xFLAG fusion constructs as labeled and proteins eluted from anti-FLAG beads were detected with a monoclonal anti-GFP antibody.

For the protein kinase genes with distinct orthologs in *S. cerevisiae*, we used the interlog approach to predict their interaction networks in *F. graminearum*. A total of 231 interactions were identified based on their yeast interlogs (Figure 8). Among them are three MAPK cascades, Fg05484-Fg09903- Fg06385, Fg00408-Fg08691-Fg09612, and Fg06326-Fg07295-Fg10313. Mutants of each MAPK pathway expressed similar phenotypes. Other predicted PK-PK interactions include the Fg04484-Fg09897, Fg10228-Fg08468, and Fg11812-Fg10313 interactions (Figure 9A). Both the Fg04484 and Fg09897 mutants grew faster in the presence of 0.7 M NaCl (Figure 5).

The same approach was used to predict the interactions of protein kinases with other proteins of *F. graminearum*. The predicted PK-protein interactome consists of 763 pairs of interactions (Figure S3). The main hubs of predicted networks include Fg08468 (Cdc28), Fg08731 (Hrr25), Fg07855 (Cdc7), Fg01271 (Cdc5), Fg10313 (Mgv1), Fg09897 (Snf1), Fg05393 (Pho85), and Fg10037 (Bud32) (Figure S3). For the two putative Cdc2/Cdc28 orthologs, only Fg08468 was included in this analysis as the representative. It was predicted to interact with 22 protein kinases and 85 other proteins. In *S. cerevisiae*, *HRR25* is involved in regulating diverse events, including vesicular trafficking, DNA repair, and chromosome segregation. FgHrr25 also was predicted to interact with 59 proteins.

To verify the predicted interactions, components of the Gpmk1 and Mgv1 MAPK pathway were selected for yeast two-hybrid and co-immunoprecipitation (co-IP) assays. In yeast two-hybrid assays, the FgSte50-Fst7, FgSte50-Fst11, Fst11-Fst7, Fst7-Gpmk1, and FgMmk2 (Fg07295)-Mgv1 interactions were confirmed by growth on SD-His (Figure 9A) and LacZ activities (Figure 9B). FgSte50 was included in this experiment because its ortholog interacts with Ste7 and Ste11 in other fungi [35], [36]. As predicted, the interaction of Fst11 with Gpmk1, Pbs2, or Hog1 was not detected. The FgMmk2-Mgv1 and FgSte7-Gpmk1 interactions were further verified by co-immunoprecipitation (co-IP) assays (Figure 9C). We also used co-IP assays to confirm the predicted interaction between *FgKIN4* (Fg11812) and Mgv1 (Figure 9C).

## Discussion

Protein kinases play key regulatory roles in various biological functions in eukaryotic organisms. To systematically characterize the kinome of *F. graminearum*, we attempted to generate PK gene knockout mutants with the split-marker approach. Among the 96 PK genes that are not essential for vegetative growth, 42 and 45 of them were found to be important for plant infection (Table 2) and sexual reproduction, respectively (Table 3). Before this study, only three protein kinase genes, Mgv1, Gpmk1, and GzSnf1, were known to be important for plant infection in *F. graminearum* [20], [21], [24]. In this study, we found that the *FgSTE11* (Fg05484) and *FgSTE7* (Fg09903) genes functioning upstream from *GPMK*1 (Fg06385) also are important for pathogenesis and sexual reproduction (Table 2; Figure 7). In yeast, the PAK kinase Ste20 functions upstream from the pheromone response pathway. While the *Fgste11* and *Fgste7* mutants had similar phenotypes with the *Gpmk1* mutant, deletion of *FgSTE20* (Fg09492) had no obvious phenotypic effect. Interestingly, deletion of *FgCLA4* (Fg06957), the only other PAK kinase gene in *F. graminearum*, resulted in a significant reduction in virulence. However, the *Fgcla4* mutant still produced ascospores and was defective in vegetative growth. These results indicate that neither of these two PAK kinases functions upstream from the Gpmk1 pathway. Orthologs of Cla4 are known to function independently of MAPK pathways in plant infection and asexual reproduction in *M. oryzae*, *U. maydis,* and *Claviceps purpurea* [37]–[39].

### The Other Two MAPK Pathways also Are Important for Plant Infection and Sexual Reproduction in *F. graminearum*

The FgBck1 (Fg06326)-FgMmk2 (Fg07295)-Mgv1 (Fg10313) MAPK cascade is orthologous to the cell integrity pathway in yeast. Similar to the *mgv1* mutant, the *Fgbck1* and *Fgmmk2* mutants formed small, whitish colonies and were defective in plant infection (Figure 7) as well as production of perithecia (Table 3). They also were defective in hyphal fusion and tended to produce wavy hyphae. In *S. cerevisiae*, the Pkc1 protein kinase C functions upstream from the yeast cell wall integrity pathway. Like Pkc1 in yeast, *FgPKC1* (Fg06268) is an essential gene in *F. graminearum*. According to the predicted PK-protein interaction networks, Mgv1 have more interacting proteins than the other two MAPKs (Figure S3). Mgv1 may regulate a number of downstream targets, such as the Mig1 and Swi4 orthologs [40], [41] in *F. graminearum*. The interactions of Mgv1 with FgMmk2 and FgKin4 were confirmed by yeast two-hybrid or co-IP assays.

The MAPK cascade orthologous to the yeast HOG pathway [42] also is well conserved in *F. graminearum.* As expected, the *Fgssk2* (Fg00408), *Fgpbs2* (Fg08691), and *Fghog1* (Fg09612) mutants were hypersensitive to hyperosmotic stresses (Figure 5). These mutants also were significantly reduced in virulence and blocked in sexual reproduction. This MAPK pathway is dispensable for plant infection in *M. oryzae* but is essential for pathogenesis in *Botrytis cinerea* and *Alternaria alternata* [43], [44]. The *Fgssk2*, *Fgpbs2*, and *Fghog1* mutants had increased sensitivities to H_2_O_2_ and reduced growth rate, which may contribute to its defects in plant infection. Interestingly, the osmoregulation pathway appears to regulate the development of aerial hyphae and fruiting bodies in *F. graminearum*. The *Fgssk2*, *Fgpbs2*, and *Fghog1* mutants were sterile and rarely produced aerial hyphae on agar pates. Hyphae of these mutants had smaller branching angles and tended to grow in parallel on the surface (Figure S4). These phenotypic effects have not been reported in other fungi. Therefore, it will be important to determine the role of this MAPK pathway in aerial hyphal growth, sexual reproduction, and pathogenesis.

### Other PK Genes Important for Plant Infection

Besides PK genes related to the MAPK and cAMP signaling pathways, over 30 PK mutants were significantly reduced in virulence (Table 2). However, many of them had severe growth defects, which may be directly responsible for reduced virulence. Among the PK mutants with a disease index less than 5, only the *Fgyak1* (Fg05418), *Fgrim15* (Fg01312), *Fgprr2* (Fg08906), and Fg04770 mutants had no significant changes in growth rate. Although these genes are conserved in filamentous ascomycetes, none of them has been reported to be important for pathogenesis in plant pathogens. The *Fgrim15* and Fg04770 mutants were reduced in the production of DON (Table S7), which is a critical virulence factor in Fusarium head blight [17]. The *Fgyak1* and *Fgprr2* mutants rarely spread from the inoculated kernels to nearby spikelets on wheat heads (disease index<1.5). The *Fgyak1* mutant had increased sensitivity to H_2_O_2_ (Table S6), 0.01% SDS, and 200 µg/ml Congo Red. In *S. cerevisiae*, Yak1 is known to regulate the stress-responsive transcription factors Hsf1 and Msn2. Fg05418 may have similar functions in *F. graminearum*. Interestingly, the *Fgprr2* mutant had increased tolerance to hyperosmotic and oxidative stresses but was reduced in virulence (Figure 5; 6).

**Figure 10:**
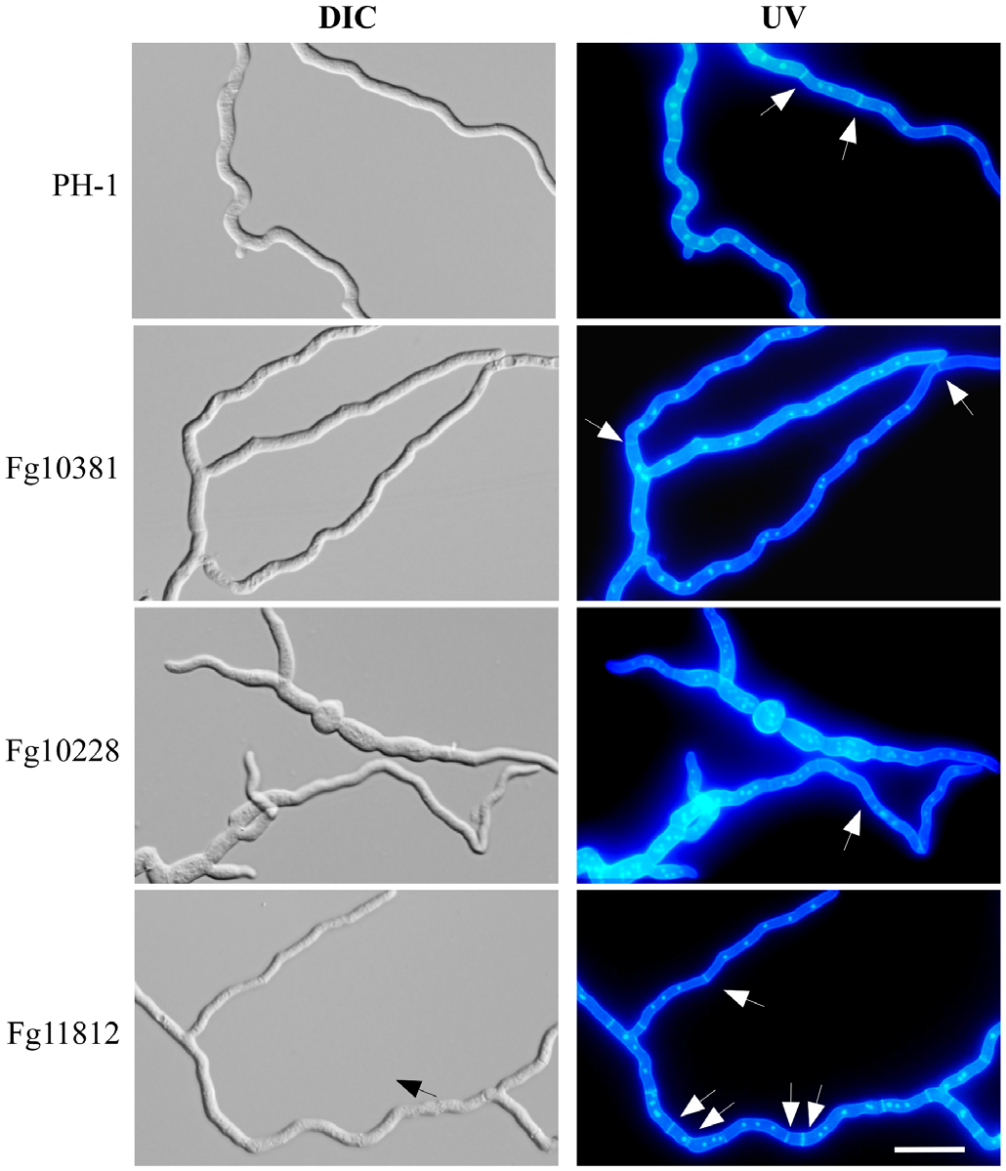
Septation defects in the Fg10228, Fg11812, and Fg10381 mutants. Germ tubes of the wild type and mutant strains were incubated at 25°C for 18 h. The same fields were observed under DIC (left) and epifluoresence (UV) microscopy. Bar = 20 µm. Arrows point to septa. Septum formation was irregular in the Fg11812 mutant, but the Fg10381 and Fg10228 mutants rarely produced septa.

The *Fgkin4* (Fg11812), *Fgsch9* (Fg00472), Fg10095, *Fgctk1* (Fg06793), *Fgcka1* (Fg03284), Fg07344, *Fgsrb10* (Fg04484), and *Fgapg1* (Fg05547) mutants had approximately 30% reduction in growth rate but a disease index less than 2.6 (Table 2). None of their orthologs except *APG1* is known to be important for plant infection in fungal pathogens. In *A. nidulans*, the Kin4-related kinase KfsA is implicated in regulating septum formation [45]. The *Fgkin4* mutant, similar to the *Fgcdc15* mutant, was defective in septum formation (Figure 10), conidiation, and sexual reproduction, further indicating that septation plays a critical role in pathogenesis and development in *F. graminearum*. In *M. oryzae*, the ortholog of *CDC15* is an important virulence factor [46]. The *Fgsch9* mutant was reduced in DON production (Table S7) and had increased sensitivity to oxidative stress (Table S6), which may be related to its reduced virulence. In yeast, Sch9 is functionally related to the PKA and TORC pathways. The role of TOR pathway is not clear in plant pathogenic fungi. The only TOR kinase gene in *F. graminearum*, Fg08133, is essential (Table 1).

Orthologs of *APG1* and autophagy are known to be important for pathogenesis in *M. oryzae* and *U. maydis* [47], [48]. It is likely that the *Fgapg1* mutant had similar defects in autophagy and infectious growth. Orthologs of *CTK1* and *SRB10* are involved in cell division in *S. cerevisiae* but have not been characterized in plant pathogenic fungi. The *Fgctk1* and *Fgsrb10* mutants all had pleiotropic defects in growth, conidiation, sexual reproduction, and plant infection (Tables 3). These two genes are likely involved in basic cellular processes, such as cell cycle or cytokinesis in *F. graminearum*.

### Protein Kinase Genes and Sexual Reproduction

Because ascospores are the primary inoculum, sexual reproduction is a critical stage of wheat scab disease cycle. A total of 45 PK genes, including all 12 members of the STE group, were found to be important for sexual reproduction. Many of these mutants also were defective in vegetative growth and plant infection. However, several PK genes, including Fg01058, Fg01347, and Fg06970 appear to play more specific or important roles in sexual reproduction. Deletion of these genes had no other significant phenotypic changes. Although deletion of Fg01058 or Fg01347 only resulted in defects in ascospore release, the Fg06970 mutant failed to produce perithecia (Table 3). Whereas Fg01058 is unique to filamentous fungi, Fg01347 is orthologous to yeast Bub1, a protein kinase phosphorylated by Cdc28 and involved in cell cycle checkpoint. Fg06970 is orthologous to yeast Psk1 and Psk2, two PAS domain-containing protein kinases that regulate protein synthesis and carbohydrate metabolism and storage [49]. Besides the Fg06970 mutant, 19 additional mutants that were blocked in perithecium formation. These mutants may be defective in female fertility or in the switch from vegetative growth to sexual reproduction.

In the budding yeast, the Smk1, Mek1, Sak1, and Ime2 kinases are required for sporulation. *F. graminearum* and other filamentous ascomyctes lack Smk1 and Mek1 orthologs. *MEK1* is a meiosis-specific protein kinase, and Smk1 MAPK regulates late stages of ascospore formation. Whereas the yeast *ime2* and *sak1* mutants are defective in sporulation, the *Fgime2* (Fg04418) and *Fgsak1* (Fg01641) mutants still produced ascospores, although the latter two mutants had pleiotropic defects. These observations indicate that ascospore formation is regulated by different mechanisms in *F. graminearum* than in *S. cerevisiae.* However, the *Fgctk1* (Fg06973), *Fgcak1* (Fg04947), *Fgkic1* (Fg05734), *Fgswe1* (Fg10228), and *Fgdbf1* (Fg08635) mutants were aborted in ascus or ascospore development (Table 3). Their orthologs also are involved in sexual reproduction in yeast. Therefore, some genetic elements are conserved between yeast and filamentous fungi for sexual reproduction.

### Essential PK Genes in *F. graminearum*

Among the 20 PK genes for which we failed to isolate knockout mutants in *F. graminearum,* most of their orthologs are essential genes in *S. cerevisiae*, *S. pombe*, or *A. nidulans* (Table 1). The *CBK1*, *KIC1*, and other RAM complex genes are essential in the wild type but not in the *ssd1* mutant of *S. cerevisiae* [50]. *F. graminearum* has an ortholog of *SSD1* (Fg07009), but deletion of *FgCBK1* or *FgKIC1* is not lethal (Table 1). Although deletion of individual genes is not lethal, the *phk1 phk2* and *ypk1 ypk2* double mutants are not viable. Fg10725 and Fg05845 are orthologs of the yeast Pkh1/Pkh2 and Ypk1/Ypk2 kinases, respectively. The downstream targets of Pkh1 and Pkh2 include Pkc1, Ypk1, and Ypk2. Ypk1 phosphorylates and down-regulates the Fpk1 kinase, a known flippase activator [51]. In *F. graminearum*, the *Fgfpk1* (Fg04382) deletion mutant was reduced in growth and had increased sensitivities to hyperosmotic and oxidative stresses.

Deletion of *PHO85*, *IRE1*, *KNS1*, or *VPS1* is not lethal in *S. cerevisiae*, but we failed to identify knockout mutants of their orthologs in *F. graminearum* (Fg05393, Fg05775, Fg06637, and Fg05306). The ortholog of *VPS1* in *A. nidulans*, *VPSA*, is involved in vacuole biogenesis. The *vpsA* mutant is viable but has poor vegetative growth [52]. In yeast, *PHO85* encodes a cyclin-dependent kinase (CDK) involved in the regulation of cellular responses to nutrient levels and environmental conditions. In *A. nidulans*, *phoA* and *phoB* are two CDKs homologous to *PHO85*. Although deletion of *phoA* or *phoB* is not lethal, the *phoA phoB* double mutant is not viable, suggesting an essential role for PhoA and PhoB in cell cycle control and morphogenesis [53]. *F. graminearum* and many other filamentous fungi have only one *PHO85* ortholog. In *U. maydis* and *C. neoformans,* deletion of the *PHO85* ortholog is lethal [54], [55].

### *F. graminearum* Uniquely Has Two Aurora Kinase Genes

Aurora kinases regulate chromosome condensation and segregation during cellular division. In *S. cerevisiae*, deletion of the *IPL1* gene is lethal. The aurora kinase gene *ark1* also is essential in *S. pombe*. Similar to the yeasts, all of the sequenced filamentous fungi, including *F. verticillioides* and *F. oxysporum*, have a single aurora kinase gene. In contrast, *F. graminearum* has two aurora kinase genes, Fg06959 and Fg02399. Whereas Fg06959 appears to be an essential gene, deletion of Fg02399 had no significant phenotypic effects other than reduced conidiation (Table 2).

### Cell Cycle- or Cytokinesis-related Kinase Genes Important for Pathogenesis

Similar to their orthologs in yeast, the *FgCDC5* (Fg01271), *FgCDC7* (Fg07855), *FgTEL1* (Fg06089), *FgMPS1* (Fg01137), *FgSGV1* (Fg07409), and *FgNIMA* (Fg09408) genes are essential in *F. graminearum*. Most likely, they have conserved functions in *F. graminearum*. While Kin3 is not essential in *S. cerevisiae*, its ortholog, NimA, is required for the regulation of mitosis in *A. nidulans* [56]. In contrast, *CDC15* is essential in yeast but deletion of its ortholog in *A. nidulans* (*SEPH*) is not lethal [57]. In *F. graminearum,* the *Fgcdc15* (Fg10381) deletion mutant was reduced in vegetative growth and conidiation. It was significantly reduced by not blocked in septation in conidia (Figure 3C) and hyphae (Figure 10).

Both Fg00677 and Fg03284 are orthologous to *CKA1*, which encodes the alpha catalytic subunit of casein kinase 2 (CK2) that is essential for cell cycle progression and proliferation in yeast. Although Fg00677 is essential in *F. graminearum,* the Fg03284 deletion mutant had no obvious defects in growth and conidiation but was significantly reduced in virulence. In *C. albicans*, the homozygous *cka2* but not *cka1* mutant has attenuated virulence in the mouse model of oropharyngeal candidiasis [58]. However, the phenotype of the *cka2* mutant can be suppressed by overexpression of *CKA1*. It is possible that Fg00677 and Fg03284 have similar functional relationship in *F. graminearum*.

In *S. cerevisiae*, *SWE1* is not essential but plays important roles in the cell cycle. The *ANKA* kinase gene, an ortholog of *SWE1*, also is involved in the regulation of septation and cell cycle checkpoint responses [59]. In *F. graminearum*, the *Fgswe1* (Fg10228) mutant was reduced in septation (Figure 10). However, it had pleiotropic defects hyphal growth, conidiation, and plant infection. In yeast, Swe1-mediated inhibition of Cdc28 is important for its checkpoint functions and pseudohyphal growth. *FgSWE1* may be important for infectious growth *in planta* in *F. graminearum*. The *SWE1* ortholog is essential in *U. maydis* [60].

### Only One of the PK Genes Related to DNA Damage Response Is Important for Plant Infection in *F. graminearum*

In *S. cerevisiae*, Rad53 is required for cell-cycle arrest in response to DNA damage. Two of the downstream targets of Rad53 are Dun1 and Dbf4. In *F. graminearum*, the *Fgrad53* (Fg00433) mutant had no obvious defects other than reduced conidiation. Whereas the *Fgdun1* (Fg07121) mutant had no detectable phenotypes, *F. graminearum*, like many other filamentous fungi, lacks a distinct ortholog of Dbf4, an essential gene required for the initiation of DNA replication. Chk1 is the other kinase functional as a DNA damage checkpoint effector in yeast and other eukaryotes [61]. Similar to the *Fgrad53* mutant, the *Fgchk1* (Fg01506) deletion mutant were normal in growth and plant infection but had increased sensitivity to UV irradiation. It appears that FgRad53 and FgChk1 kinases are important for DNA damage repair but dispensable for pathogenesis in *F. graminearum*.

In yeast, both Rad53 and Chk1 are phosphorylated by Mec1, an essential gene involved in the cell cycle checkpoint control in response to DNA damage. In *F. graminearum*, deletion of *FgMEC1* (Fg13318) was not lethal but the *Fgmec1* mutant was significantly reduced in virulence. It also was reduced in growth rate, conidiation, had increased sensitivity to H_2_O_2_. In *A. nidulans*, the AtmA and UvsB kinases, orthologs of Tel1 and Mec1, also are functionally related in regulating DNA damage responses and act upstream from the ChkA and ChkB check point kinases [62].

### PK Genes with No Distinct Orthologs in *S. cerevisiae*

Among the 28 *F. graminearum* PK genes that lack distinct orthologs in *S. cerevisiae*, four of them have orthologs in *S. pombe* (Table S8). Whereas the function of *ppk23* is not clear, the intracellular gradient of Pom1 is used as the sensor for cell length in *S. pombe* [63]. In *F. graminearum*, the *Fgppk23* (Fg05406) mutant had no phenotype other than reduced conidiation. The *Fgpom1* (Fg10095) mutant was defective in plant infection, DON synthesis, and sexual reproduction although it was only slightly reduced in conidiation and vegetative growth. Although *prp4* and *sid1* are essential genes in *S. pombe*, the *Fgsid1* (Fg07344) and *Fgprp4* (Fg04053) mutants were viable but displayed pleiotrophic defects. The *Fgprp4* mutant has severe growth defects. Prp4 is involved in spliceosome functions in *S. pombe* [64]. Interestingly, the *Fgprp4* mutant was unstable. We had identified over a dozen spontaneous suppressor mutants with faster growth rate. Further characterization of the *Fgprp4* mutant and suppressor mutations will be useful to determine the role this protein kinase in RNA splicing and fungal pathogenesis.

For 15 of the other 24 PK genes that appear to be specific for filamentous fungi, their knockout mutants had no obvious phenotypic changes (Table S7). Some of them may be not true PK genes. Among the rest 9 genes, deletion of Fg09150 resulted in approximately 80% reduction in conidiation but had no other detectable phenotypic effect. In contrast, the Fg01058 mutant was defective only in ascospore morphology and release, suggesting that these two PK genes have specific functions during asexual and sexual reproduction, respectively. The Fg00792, Fg01559, Fg02488, and Fg06420 mutants were slightly reduced in DON production but had no significant defects in plant infection. Therefore, Fg00362, Fg03146, and Fg04770 are the only three PK genes that are absent in the yeasts but important for plant infection in *F. graminearum* (Table S8). The Fg00362 mutant grew poorly (Table 2). *POD-6*, an ortholog of Fg00362, has been functionally characterized in *N. crassa* [10]. It interacts with *COT-1* and plays a critical role in hyphal growth. Their orthologs likely have conserved functions in *F. graminearum* because the *Fgpod6* and *Fgcot1* mutants had the same growth defects (Figure S2). In contrast, the Fg03146 and Fg04770 mutants had no obvious defects in growth. Orthologs of Fg03146 and Fg04770 have not been characterized in filamentous fungi. It will be important to further characterize these two novel fungal virulence factors.

## Materials and Methods

### Identification of PK Genes

Protein sequences of *F. graminearum* were searched against the Kinomer v.1.0 HMM library using the HMMSCAN program from the HMM software suite HMMer (version 3.0 for windows) to identify and classify protein kinases as described [3], [65]. The cut off value was set to 20. We also searched for additional putative PK genes that are predicted by the Broad Institute (www.broadinstitute.org/annotation/genome/fusarium_graminearum) or MIPS (mips.helmholtz-muenchen.de/genre/proj/FGDB) to contain the protein kinase domain (Pkinase, PF00069). Phylogenetic analysis was conducted with *MEGA* version 5 [66]. The catalytic domain sequences were aligned with COBALT [67] and trimmed with trimAl [68]. The maximum likelihood phylogeny tree was visualized using Interactive Tree Of Life Version 1.9 (http://itol.embl.de/#).

### Generation of the Knockout Mutants

The split-marker approach [19] was used to generate the gene replacement constructs for the PK genes. The primers used to amplify the flanking sequences for each gene are available at fgkinome.nwsfau.edu.cn. The resulting PCR products were transformed into protoplasts of the wild-type strain PH-1 [69] as described [17], [20]. Hygromycin B (Calbiochem, La Jolla, CA) was added to a final concentration of 250 µg/ml for transformant selection. Putative knockout mutants identified by screening with primers F5 and R6 were further analyzed by PCR with primers F7 and H856R or primers H855F and R8 to confirm the gene replacement events (Figure S1). All of the mutants generated in this study were preserved in 15% glycerol at −80°C.

### Assays for Growth and Conidiation Defects

Colony morphology and growth rate were assayed with potato dextrose agar (PDA) cultures grown at 25°C for three days. Conidiation was assayed with 5-day-old CMC cultures as described [20], [70]. Conidium morphology was examined and photographed with an Olympus BX-51 microscope. For assaying conidium germination and germ tube growth, freshly harvested conidia were cultured in liquid YEPD medium for 12 h. Slab cultures grown on a thin layer of complete medium (CM) for 36 h were examined for defects in hyphal tip growth and branching [20], [70].

### Assays for Defects in Sexual Reproduction and Stress Responses

Aerial hyphae of 7-day-old carrot agar cultures were pressed down with 300 µl of sterile 0.1% Tween 20. Perithecium formation and cirrhi production were assayed after incubation at 25°C for 2 weeks. For mutants that formed perithecia but failed to produce cirrhi 3-4 weeks after fertilization, at least 10 perithecia were examined for ascospores and ascogenous hyphae. For assaying sensitivities to various stresses, vegetative growth was assayed on PDA plates with 0.7 M NaCl, 0.05% H_2_O_2,_ 0.01% SDS, or 200 µg/ml Congo Red [71].

### Plant Infection and DON Production Assays

Conidia harvested from 5-day-old CMC cultures were resuspended to 10^6^ spores/ml. Flowering wheat heads of cultivar Xiaoyan 22 were drop-inoculated with 10 µl of conidium suspensions at the fifth spikelet from the base of the inflorescence [72], [73]. After the inoculation, wheat heads were capped with a plastic bag for 48 h to maintain the moisture. Spikelets with typical symptoms were examined 14 days post-inoculation (dpi). Diseased wheat kernels were pooled to assay for DON production as described [20]. For stalk rot assays, 8-week-old corn plants of cultivar Pioneer 2375 were inoculated as described [70], [74] and assayed for symptoms 14 dpi. Infection assays with corn silks were conducted as described [20].

### Prediction of Protein-Protein Interactions (PPIs)

The protein-protein interaction (PPI) networks of *S. cerevisiae* were downloaded from the Database of Interacting Proteins (DIP, dip.doe-mbi.ucla.edu/dip) and SGD (www.yeastgenome.org). Orthologous pairs of *F. graminearum* and *S. cerevisiae* genes were obtained from the Inparanoid database [75] and by BlastP searches. To strengthen the reliability of predicted interactions, the bit score cut off value was set to 200. The predicted PPI interaction map was generated with the Cytoscape program [76].

### Yeast Two-Hybrid Assays

Protein-protein interactions were assayed with the Matchmaker yeast two-hybrid system (Clontech, Mountain View, CA). ORFs of the *GPMK1*, *FgSTE50* (Fg04101), and *FgSTE7* (Fg09903) were amplified from first-strand cDNA of PH-1 and cloned into pGBK7 (Clontech) as the bait constructs. For the *FgSTE11* (Fg05484), *FgHOG1* (Fg09612), and *FgPBS2* (Fg08691) genes, their ORFs were amplified and cloned into pGADT7 as the prey constructs. Prey constructs also were generated for the *GPMK1* and *FgSTE50* genes. The resulting bait and prey vectors were co-transformed in pairs into yeast strain AH109 (Clontech). The Leu^+^ and Trp^+^ transformants were isolated and assayed for growth on SD-Trp-Leu-His medium and galactosidase activities with filter lift assays as described [77]. The positive and negative controls were provided in the Matchmaker Library Construction & Screening Kits (Clontech).

### Co-immunoprecipitation (co-IP) Assays

The *GPMK1* and *MGV1* genes were amplified and cloned into pDL2 by the yeast gap repair approach [78], [79] to generate the 3xFLAG fusion constructs. Similar approaches were used to generate the GFP fusion constructs for the *FgMMK2* (Fg07295), *FgKIN4* (Fg11812),and *FgSTE7* (Fg09903) genes with the pFL3 vector [80]. The resulting fusion constructs were verified by DNA sequencing and transformed in pairs into PH-1. Transformants expressing pairs of fusion constructs were confirmed by western blot analysis. For co-IP assays, total proteins were isolated and incubated with the anti-FLAG M2 beads as described [81]. Proteins eluted from beads were analyzed by western blot detection with a monoclonal anti-GFP (Roche, Indianapolis, IN) antibody.

## Supporting Information

Figure S1 PCR primers used for the generation and identification of gene replacement mutants. The arrows indicate the directions of the primers.
(EPS)

Figure S2 Growth defects of the *Fgcot1* (Fg01188), *Fgpod6* (Fg00362), and *Fgprp4* (Fg04053) mutants. Colony morphology (colony), hyphal growth on complete medium (CM) slab agar (agar), and hyphae cultured in liquid CM (liquid).
(EPS)

Figure S3 Predicted interactions of protein kinases with other proteins of *F. graminearum*. Orthologs of interacting proteins in *S. cerevisiae* were identified and used for the prediction of protein kinase (PK)-protein interactions. The PK-protein interaction map was generated with Cytoscape.
(EPS)

Figure S4 Defects of the osmoregulation MAPK pathway mutants in aerial hyphal growth and branching.
(EPS)

Table S1 Putative protein kinase genes in *Fusarium graminearum.*
(DOC)

Table S2 Protein kinase genes that are closely linked to each other in *F. graminearum*.
(DOC)

Table S3 Expression profiles of protein kinase genes during barley infection, sexual development, and conidium germination.
(DOC)

Table S4 The Pearson correlation efficient between major phenotypes.
(DOC)

Table S5 Mutants with defects in germ tube (GT) growth.
(DOC)

Table S6 Mutants with altered responses to stresses.
(DOC)

Table S7 Kinase mutants with 30% reduction in DON production.
(DOC)

Table S8 Mutant phenotypes of 28 *F graminearum* PK genes with no distinct orthologs in *S. cerevisiae*.
(DOC)
