# Supplementary material for: Functional Analysis of the Kinome of the Wheat Scab Fungus Fusarium graminearum
Source: PLoS Pathog. 2011 Dec 22;7(12):e1002460. doi: 10.1371/journal.ppat.1002460 (PMC3245316; doi:10.1371/journal.ppat.1002460)
Supplement: Table S1 — Putative protein kinase genes in Fusarium graminearum. (DOC) [file ppat.1002460.s005.doc]

**Table S1. Putative Protein Kinase Genes in *Fusarium graminearum***

| **Group** | ***Fusarium graminearum* ^a^** | ***Saccharomyces cerevisiae* ^d^** |
| --- | --- | --- |
| **AGC** |  |  |
|  | **Fg05845** | *YPK1, YPK2* |
|  | Fg00472 **^b^** | *SCH9* |
|  | **Fg06959** | ***IPL1*** |
|  | Fg02399 | ***IPL1*** |
|  | Fg01312 | *RIM15* |
|  | Fg01188 **^b^** | ***CBK1*** |
|  | Fg08635 | *DBF2, DBF20* |
|  | **Fg10725** | *PKH1, PKH2* |
|  | Fg08729 | *TPK1,* |
|  | Fg07251 **^b, c^** | *TPK2, TPK3* |
|  | **Fg09660** | ***PKC1*** |
|  | Fg04382 | *FPK1* |
|  | Fg00469 | *YPK3* |
|  | Fg08631 | Ypk2-like |
|  | Fg01058 **^b, c^** |  |
| **CAMK** |  |  |
|  | Fg06878 | *CMK1,* |
|  | Fg00337 | *CMK2* |
|  | **Fg01271** **^b^** | ***CDC5*** |
|  | Fg07520 | *IKS1* |
|  | Fg09513 | *SCY1* |
|  | Fg10196 | Scy1-like |
|  | **Fg05775** | *IRE1* |
|  | Fg06832 | *ENV7* |
|  | Fg00786 | *RCK1, RCK2* |
|  | Fg06940 **^b^** | *TOS3* |
|  | Fg01641 | *SAK1* |
|  | Fg01506 | *CHK1* |
|  | Fg09274 | *KIN1, KIN2* |
|  | Fg11812 **^b, c^** | *KIN4, FRK1* |
|  | Fg05764 | *YPL150W* |
|  | Fg06970 | *PSK2, PSK1* |
|  | Fg09897 | *SNF1* |
|  | Fg08701 **^c^** | *GIN4 like* |
|  | Fg01842 | *PRR1* |
|  | Fg06939 **^b^** | *SAT4* (*HAL4*) |
|  | Fg06206 | *HRK1, RTK1* |
|  | Fg08906 **^c^** | *PRR2* |
|  | Fg00433 **^c^** | ***RAD53*** |
|  | Fg07121 | *DUN1* |
|  | Fg12149 **^b^** | *KSP1* |
|  | **Fg04054** | *VHS1, SKS1* |
|  | Fg05586 | *PRK1, ARK1, AKL1* |
|  | Fg07816 |  |
|  | Fg02838 |  |
|  | Fg06420 |  |
|  | Fg00792 |  |
|  | Fg01559 **^b^** |  |
|  | Fg05549 |  |
| **CK1** |  |  |
|  | Fg10066 | *YCK1/2/3* |
|  | **Fg08731** | ***HRR25*** |
| **CMGC** |  |  |
|  | Fg10228 **^b, c^** | *SWE1* |
|  | **Fg07423** | ***KIN28*** |
|  | Fg06793 **^b^** | *CTK1* |
|  | **Fg05306** | *VPS15* |
|  | Fg08468 **^c^** | ***CDC28*** *(CDC2)* |
|  | Fg03132 **^c^** | ***CDC28*** *(CDC2)* |
|  | Fg04947 **^c^** | ***CAK1*** |
|  | **Fg07409** | ***SGV1*** |
|  | **Fg05393** | *PHO85* |
|  | Fg04484 **^c^** | *SRB10* |
|  | **Fg00677** | ***CKA1*** |
|  | Fg03284 | *CKA2* |
|  | **Fg06637 ^b^** | *KNS1* |
|  | Fg05418 | *YAK1* |
|  | Fg07329 **^c^** | *GSK3* (*RIM11*), *MRK1* |
|  | Fg06385 | *FUS3, KSS1* |
|  | Fg09612 **^c^** | *HOG1* |
|  | Fg10313 | *SLT2* |
|  | Fg04418 | *IME2* |
|  | Fg02795 **^c^** | *SKY1* |
|  | Fg05406 |  |
|  | Fg10095 |  |
|  | Fg04053 **^b, c^** |  |
|  | Fg02488 **^b^** |  |
|  | Fg03146 **^b^** |  |
|  | Fg00132 |  |
| **STE** |  |  |
|  | Fg08691 **^c^** | *PBS2* |
|  | Fg07295 **^c^** | *MKK1, MKK2* |
|  | Fg09903 | *STE7* |
|  | Fg09492 | *STE20* |
|  | Fg06957 | *CLA4, SKM1* |
|  | Fg07344 **^c^** |  |
|  | Fg05734 | *SPS1,* ***KIC1*** |
|  | Fg10381 **^c^** | ***CDC15*** |
|  | Fg00408 | *SSK2, SSK22* |
|  | Fg05484 | *STE11* |
|  | Fg06326 **^c^** | *BCK1* |
|  | Fg00362 **^c^** |  |
| **Others** |  |  |
|  | Fg05547 **^b^** | *ATG1* (*APG1*) |
|  | Fg10037 | *BUD32* |
|  | Fg01347 | *BUB1* |
|  | Fg05135 | *GCN2* |
|  | **Fg09408** | *KIN3* |
|  | **Fg07855** | ***CDC7*** |
|  | **Fg01137** | ***MPS1*** |
|  | Fg12887 **^b^** |  |
|  | Fg09150 |  |
|  | Fg12132 |  |
|  | Fg04770 **^b^** |  |
|  | Fg11614 |  |
|  | Fg07742 |  |
|  | Fg07745 |  |
|  | Fg02153 |  |
|  | Fg13509 |  |
|  | Fg10591 |  |
|  | Fg03499 **^b^** |  |
|  | Fg13944 |  |
|  | Fg07812 |  |
| **PDHK** |  |  |
|  | Fg07381 | *PKP2* |
|  | Fg01963 | *PKP1* |
|  | Fg04416 |  |
| **PIKK** |  |  |
|  | Fg13318 | ***MEC1*** |
|  | **Fg05519** | ***TEL1*** |
|  | **Fg08133** **^b^** | *TOR1,* ***TOR2*** |
|  | **Fg06089** | ***TRA1*** |
| **RIO** |  |  |
|  | **Fg06502** | ***RIO1****,* ***RIO2*** |

**^a^** Gene numbers of predicted PK genes of version 3 were abbreviated by replacing FGSG_ with Fg. For example, FGSG_06089.3 was abbreviated as Fg06089.

**^b^** Genes with annotation problems in automated annotations.

**^c^** Knockout mutants confirmed by Southern blot analyses

**^d^** No distinct orthologs of *DBF4*, *SMK1*, *MEK1, ELM1, ALK1, ALK2, PTK1, PTK2, NPR1, KCC4, GIN4, HSL1, ISR1, YGR052W, YLR253W, YMR291W, HAL5, KKQ8,* and *NNK1* in *F. graminearum*.

**^e^** *F. graminearum* lacks PKs belonging to the RGC, TK, TKL, and Alpha groups

**^f^** Essential genes in *F. graminearum* or *S. cerevisiae* are in bold.
