# Supplementary material for: Functional Analysis of the Kinome of the Wheat Scab Fungus Fusarium graminearum
Source: PLoS Pathog. 2011 Dec 22;7(12):e1002460. doi: 10.1371/journal.ppat.1002460 (PMC3245316; doi:10.1371/journal.ppat.1002460)
Supplement: Table S2 — Protein kinase genes that are closely linked to each other in F. graminearum. (DOC) [file ppat.1002460.s006.doc]

**Table S2. Protein Kinase Genes That Are Closely Linked to Each Other in *F. graminearum*.**

| ***F. graminearum* (V3)** | ***F. verticillioides* (V3)** | ***F. oxysporum* (V2)** | ***Neurospora crassa* (V5)** | ***Magnaporthe oryzae* (V7)** | ***Aspergillus nidulans* (V1)** |
| --- | --- | --- | --- | --- | --- |
| FGSG_06939 **^a^**  FGSG_06940 | FVEG_08257  FVEG_08256 | FOXG_01880  FOXG_01878 | NCU_06179  NCU_06177 | MGG_06420  MGG_06421 | ANID_08830  ANID_08827 |
| FGSG_04053 **^b^**  FGSG_04054 | FVEG_10226  FVEG_10227 | FOXG_11594  FOXG_11593 | NCU_10853  NCU_04990 | MGG_12944  MGG_05074 | ANID_04936  ANID_04935 |
| FGSG_08729 **^c^**  FGSG_08731 | FVEG_01931  FVEG_01929 | FOXG_03067  FOXG_03065 | NCU_00682  NCU_00685 | MGG_02832  MGG_02829 | ANID_04717  ANID_04563 |
| FGSG_00469 **^d^**  FGSG_00472 | FVEG_00928  FVEG_00931 | FOXG_00585  FOXG_00582 | NCU_03197  NCU_03200 | MGG_01260  MGG_14773 | ANID_04980  ANID_04238 |
| FGSG_06957 **^e^**  FGSG_06959 | FVEG_08235  FVEG_08233 | FOXG_01858  FOXG_01856 | NCU_00406  NCU_00108 | MGG_06320  MGG_00479 | ANID_08836  ANID_05815 |
| FGSG_05547  FGSG_05549 | FVEG_06949  FVEG_06947 | FOXG_09345  FOXG_09343 | NCU_00188  NCU_06486 | MGG_06393  MGG_17341 | ANID_01632  ANID_07737 |
| FGSG_04416  FGSG_04418 | FVEG_11246  FVEG_11244 | FOXG_13817  FOXG_13813 | NCU_06760  NCU_01498 | MGG_07161  MGG_01822 | ANID_06207  ANID_06243 |

**^a^** Orthologs of FGSG_06939 and FGSG_06940 are closely linked all the filamentous fungi examined.

**^b^** Orthologs of FGSG_04053 and FGSG_04054 are closely linked in *F. verticillioides, F. oxysporum*, and *A. nidulans* but not in *M. oryzae* and *N. crassa*.

**^c^** The linkage between Fg08729 and Fg08731 is conserved in *F. verticillioides*, *F. oxysporum*, *M. oryzae*, and *N. crassa* but not in *A. nidulans*.

**^d^** Orthologs of Fg00469 and Fg00472 are closely linked in *F. verticillioides*, *F. oxysporum*, and *N. crassa* but not in *M. oryzae* and *A. nidulans*.

**^e^** The linkage between Fg06957 and Fg06959, Fg05547 and Fg05549, or FGSG_04416 and FGSG_04418, is only conserved in the three sequenced *Fusarium* species. Their orthologs in other filamentous fungi are not closely linked.
