# Supplementary material for: Functional Analysis of the Kinome of the Wheat Scab Fungus Fusarium graminearum
Source: PLoS Pathog. 2011 Dec 22;7(12):e1002460. doi: 10.1371/journal.ppat.1002460 (PMC3245316; doi:10.1371/journal.ppat.1002460)
Supplement: Table S3 — Expression profiles of protein kinase genes during barley infection, sexual development, and conidium germination. (DOC) [file ppat.1002460.s007.doc]

**Table S3. Expression Profiles of Protein Kinase Genes during Barley Infection, Sexual Development, and Conidium Germination.**

| **Gene** | **FG1: Infection of Morex barley spikes** | | | | | **FG5: *in vitro* sexual development** | | | | | **FG7: Conidium germination** | | |
| --- | --- | --- | --- | --- | --- | --- | --- | --- | --- | --- | --- | --- | --- |
|  | 24 | 48 | 72 | 96 | 144 | 24 | 48 | 72 | 96 | 144 | 2 | 8 | 24 |
| Fg05845 | 0.9 | 0.7 | 0.7 | 0.7 | 0.6 | 1.1 | 1.0 | 1.4 | 2.0 | 1.4 | 1.0 | 1.1 | 1.0 |
| Fg00472 | 1.3 | 1.7 | 2.6 | 3.1 | 5.9 | 1.1 | 0.6 | 0.4 | 0.4 | 0.5 | 2.5 | 1.3 | 1.3 |
| Fg06959 | 1.0 | 1.0 | 0.9 | 0.7 | 0.6 | 0.9 | 1.0 | 1.1 | 1.3 | 0.9 | 1.0 | 1.1 | 1.4 |
| Fg02399 | 1.6 | 0.9 | 0.9 | 0.9 | 0.7 | 1.0 | 2.9 | 3.9 | 3.3 | 2.9 | 1.0 | 1.1 | 1.1 |
| Fg01347 | 1.1 | 1.1 | 0.9 | 0.8 | 0.8 | 0.7 | 0.7 | 0.7 | 0.6 | 0.7 | 4.1 | 2.2 | 1.0 |
| Fg01312 | 0.9 | 0.8 | 0.9 | 0.8 | 1.0 | 1.5 | 1.6 | 1.4 | 1.3 | 1.8 | 0.5 | 0.8 | 1.1 |
| Fg01188 | 0.8 | 1.1 | 1.3 | 1.9 | 1.9 | 1.0 | 0.9 | 1.4 | 1.9 | 1.3 | 1.7 | 2.0 | 1.2 |
| Fg08635 | 1.3 | 1.1 | 1.1 | 1.1 | 1.1 | 1.9 | 0.9 | 0.6 | 0.8 | 4.1 | 4.1 | 6.0 | 2.1 |
| Fg10725 | 1.2 | 1.2 | 1.2 | 1.2 | 1.7 | 0.7 | 0.8 | 0.6 | 0.6 | 0.9 | 1.0 | 0.5 | 0.6 |
| Fg08729 | 1.1 | 1.1 | 1.0 | 0.9 | 1.1 | 0.6 | 1.2 | 0.9 | 0.6 | 0.7 | 0.8 | 0.5 | 0.2 |
| Fg07251 | 0.9 | 0.9 | 0.8 | 0.9 | 0.9 | 1.3 | 1.1 | 1.0 | 1.0 | 1.0 | 1.0 | 1.0 | 1.0 |
| Fg09660 | 0.9 | 1.1 | 1.7 | 2.2 | 4.7 | 1.1 | 1.1 | 0.9 | 1.0 | 1.2 | 1.2 | 2.2 | 2.1 |
| Fg04382 | 0.9 | 0.9 | 0.9 | 0.9 | 0.8 | 0.8 | 1.0 | 0.6 | 0.4 | 0.5 | 0.5 | 0.4 | 0.7 |
| Fg00469 | 0.9 | 0.8 | 0.8 | 0.7 | 0.7 | 1.0 | 1.2 | 1.2 | 1.1 | 1.6 | 1.0 | 0.2 | 0.5 |
| Fg08631 | 0.9 | 0.9 | 0.9 | 1.0 | 1.1 | 1.1 | 1.5 | 0.6 | 0.4 | 0.9 | 0.5 | 0.3 | 0.3 |
| Fg01058 | 1.0 | 1.1 | 0.7 | 0.8 | 0.7 | 0.9 | 0.9 | 2.0 | 2.9 | 75.1 | 1.8 | 0.8 | 0.8 |
| Fg06878 | 1.5 | 2.2 | 5.2 | 5.8 | 8.6 | 0.9 | 1.0 | 1.1 | 0.5 | 0.9 | 0.4 | 1.1 | 1.9 |
| Fg00337 | 0.8 | 1.0 | 1.0 | 1.3 | 1.8 | 2.1 | 1.6 | 1.6 | 1.1 | 1.3 | 0.5 | 0.6 | 0.3 |
| Fg00786 | 1.1 | 1.0 | 0.9 | 1.0 | 1.8 | 0.4 | 0.5 | 0.5 | 0.4 | 0.5 | 0.2 | 0.3 | 0.7 |
| Fg06940 | 0.9 | 0.9 | 0.9 | 0.9 | 1.1 | 2.2 | 2.2 | 1.8 | 1.4 | 1.2 | 0.6 | 0.8 | 0.8 |
| Fg01506 | 1.0 | 0.9 | 0.8 | 0.7 | 0.6 | 1.2 | 0.8 | 0.7 | 1.0 | 1.9 | 1.4 | 3.7 | 1.6 |
| Fg09274 | 1.1 | 1.0 | 1.3 | 1.5 | 2.0 | 1.2 | 1.0 | 1.1 | 1.0 | 1.0 | 0.8 | 0.8 | 0.8 |
| Fg11812 | 0.8 | 0.8 | 0.8 | 0.8 | 1.0 | 0.6 | 0.7 | 0.5 | 0.5 | 0.6 | 0.8 | 0.5 | 0.5 |
| Fg05764 | 1.1 | 0.9 | 1.0 | 0.8 | 0.9 | 0.8 | 1.0 | 0.7 | 0.7 | 1.2 | 1.3 | 0.8 | 0.7 |
| Fg06970 | 0.7 | 0.8 | 0.9 | 1.2 | 1.8 | 1.3 | 1.1 | 1.2 | 1.4 | 1.8 | 0.7 | 0.5 | 0.5 |
| Fg09897 | 1.1 | 1.2 | 1.5 | 2.0 | 3.2 | 1.4 | 1.0 | 1.0 | 0.8 | 0.8 | 0.7 | 2.0 | 2.1 |
| Fg08701 | 1.0 | 0.9 | 0.7 | 0.7 | 0.6 | 20.3 | 18.5 | 14.3 | 9.9 | 11.9 | 0.2 | 0.2 | 0.1 |
| Fg01842 | 1.0 | 0.9 | 0.8 | 0.7 | 0.7 | 1.1 | 1.2 | 0.8 | 0.9 | 1.6 | 0.2 | 0.1 | 0.2 |
| Fg06939 | 0.8 | 1.2 | 1.2 | 1.6 | 2.3 | 1.0 | 0.9 | 1.4 | 1.3 | 1.0 | 1.1 | 0.6 | 0.7 |
| Fg06206 | 1.1 | 1.0 | 1.1 | 1.1 | 1.3 | 1.0 | 1.1 | 1.0 | 0.9 | 1.5 | 0.5 | 0.5 | 0.9 |
| Fg08906 | 1.0 | 0.9 | 1.0 | 1.1 | 1.2 | 1.7 | 1.7 | 1.3 | 0.8 | 2.1 | 1.6 | 1.4 | 2.0 |
| Fg00433 | 1.0 | 1.0 | 0.9 | 0.9 | 1.1 | 1.1 | 0.9 | 0.9 | 1.0 | 2.8 | 1.2 | 0.7 | 0.7 |
| Fg07121 | 1.0 | 0.9 | 0.9 | 1.0 | 1.2 | 1.0 | 0.9 | 0.9 | 0.8 | 1.1 | 0.8 | 0.7 | 0.9 |
| Fg12149 | 0.9 | 1.2 | 2.0 | 2.8 | 8.2 | 0.5 | 1.1 | 0.4 | 0.3 | 0.6 | 0.4 | 0.8 | 0.6 |
| Fg05547 | 0.8 | 0.8 | 0.7 | 0.7 | 0.7 | 2.1 | 1.7 | 1.2 | 1.1 | 1.2 | 0.4 | 0.6 | 0.9 |
| Fg04054 | 1.0 | 0.9 | 1.0 | 1.1 | 1.5 | 0.7 | 1.4 | 0.5 | 0.5 | 1.2 | 2.1 | 1.3 | 0.7 |
| Fg07816 | 1.0 | 1.2 | 1.1 | 1.3 | 1.4 | 0.7 | 0.8 | 1.2 | 0.8 | 0.3 | 2.0 | 1.9 | 4.5 |
| Fg05549 | 1.0 | 0.9 | 0.9 | 0.9 | 0.8 | 1.4 | 1.9 | 1.8 | 3.1 | 15.5 | 1.5 | 1.7 | 1.5 |
| Fg10066 | 0.8 | 1.0 | 1.2 | 1.2 | 1.2 | 0.6 | 0.7 | 0.2 | 0.2 | 0.7 | 0.8 | 0.8 | 0.7 |
| Fg08731 | 1.2 | 2.2 | 5.1 | 6.8 | 15.4 | 0.5 | 0.9 | 0.3 | 0.2 | 1.0 | 1.2 | 0.9 | 1.1 |
| Fg07855 | 1.0 | 1.0 | 1.1 | 1.0 | 1.0 | 1.6 | 1.3 | 1.3 | 1.3 | 2.2 | 1.4 | 0.6 | 0.2 |
| Fg07423 | 1.2 | 1.1 | 0.9 | 0.9 | 0.8 | 1.1 | 1.0 | 1.6 | 2.0 | 2.4 | 0.7 | 0.5 | 0.2 |
| Fg06793 | 1.1 | 1.0 | 1.0 | 1.0 | 1.0 | 1.0 | 1.2 | 1.2 | 0.8 | 1.1 | 0.7 | 0.4 | 0.3 |
| Fg04947 | 1.0 | 0.8 | 0.8 | 0.7 | 0.7 | 1.0 | 1.1 | 1.1 | 1.9 | 31.5 | 0.8 | 1.0 | 0.9 |
| Fg07409 | 0.9 | 0.8 | 0.6 | 0.8 | 0.7 | 0.8 | 1.0 | 0.6 | 0.8 | 1.4 | 1.1 | 0.5 | 0.4 |
| Fg05393 | 1.2 | 1.3 | 1.4 | 1.9 | 3.5 | 1.4 | 0.9 | 1.5 | 0.4 | 0.6 | 1.6 | 1.4 | 1.1 |
| Fg04484 | 0.9 | 1.0 | 0.8 | 0.9 | 0.9 | 0.9 | 1.9 | 1.3 | 1.1 | 1.6 | 0.3 | 0.1 | 0.1 |
| Fg00677 | 1.2 | 1.4 | 3.2 | 3.4 | 9.2 | 1.1 | 0.9 | 0.5 | 0.4 | 0.7 | 1.8 | 1.1 | 0.7 |
| Fg03284 | 1.0 | 0.9 | 0.9 | 1.0 | 1.0 | 2.1 | 2.1 | 2.6 | 1.1 | 0.5 | 0.8 | 1.6 | 2.5 |
| Fg06637 | 1.2 | 1.0 | 1.1 | 1.4 | 2.9 | 0.9 | 1.2 | 1.0 | 0.6 | 0.9 | 0.5 | 0.3 | 0.6 |
| Fg05418 | 1.0 | 1.0 | 0.8 | 0.9 | 1.1 | 0.9 | 0.9 | 0.4 | 0.4 | 1.0 | 1.0 | 0.8 | 0.6 |
| Fg07329 | 1.2 | 1.6 | 2.2 | 2.5 | 6.0 | 0.7 | 1.2 | 0.4 | 0.3 | 0.9 | 1.8 | 2.0 | 1.9 |
| Fg06385 | 0.9 | 1.9 | 3.0 | 4.5 | 7.8 | 1.2 | 1.1 | 0.9 | 1.0 | 1.0 | 1.1 | 2.1 | 1.0 |
| Fg09612 | 1.0 | 1.1 | 1.0 | 1.3 | 3.0 | 0.3 | 0.3 | 0.4 | 0.4 | 0.7 | 6.4 | 4.2 | 3.0 |
| Fg10313 | 1.1 | 1.8 | 2.0 | 2.3 | 4.0 | 1.2 | 1.3 | 1.0 | 1.0 | 1.0 | 0.1 | 1.0 | 0.7 |
| Fg04418 | 0.9 | 0.8 | 0.9 | 0.9 | 1.1 | 0.6 | 0.6 | 0.4 | 0.5 | 0.4 | 0.8 | 2.0 | 1.2 |
| Fg02795 | 1.3 | 2.4 | 6.6 | 7.1 | 11.3 | 1.2 | 1.0 | 1.4 | 1.4 | 0.8 | 1.2 | 0.8 | 0.8 |
| Fg01137 | 1.0 | 0.8 | 0.7 | 0.8 | 0.6 | 1.8 | 0.7 | 0.4 | 0.6 | 1.5 | 1.5 | 3.1 | 1.2 |
| Fg05406 | 1.0 | 0.8 | 0.7 | 0.7 | 0.7 | 1.0 | 0.8 | 0.6 | 0.8 | 1.2 | 1.4 | 0.9 | 0.8 |
| Fg10095 | 1.0 | 1.0 | 1.1 | 0.9 | 1.3 | 1.3 | 1.0 | 0.6 | 0.5 | 0.8 | 1.1 | 0.5 | 0.3 |
| Fg04053 | 1.1 | 0.9 | 0.8 | 0.7 | 0.7 | 0.9 | 0.9 | 0.9 | 1.3 | 1.1 | 1.0 | 1.1 | 1.2 |
| Fg02488 | 0.8 | 0.8 | 1.0 | 0.8 | 0.8 | 2.3 | 0.9 | 1.2 | 0.7 | 0.7 | 0.1 | 0.0 | 0.0 |
| Fg07812 | 0.8 | 0.8 | 0.6 | 0.6 | 0.4 | 1.4 | 1.9 | 2.2 | 1.5 | 1.8 | 0.6 | 0.5 | 0.5 |
| Fg03146 | 1.2 | 0.9 | 0.9 | 0.8 | 0.7 | 1.1 | 0.9 | 0.7 | 0.8 | 0.7 | 1.3 | 1.3 | 1.1 |
| Fg00132 | 1.3 | 0.8 | 0.8 | 0.7 | 0.6 | 1.0 | 1.1 | 1.0 | 1.0 | 0.9 | 1.0 | 1.0 | 1.0 |
| Fg03132 | 1.1 | 1.2 | 1.3 | 1.5 | 1.8 | 0.5 | 0.3 | 0.2 | 0.2 | 0.7 | 11.1 | 8.9 | 4.1 |
| Fg08468 | 1.2 | 1.3 | 1.2 | 0.9 | 0.8 | 0.8 | 0.8 | 0.5 | 0.6 | 1.5 | 2.9 | 2.0 | 1.8 |
| Fg08691 | 1.1 | 1.0 | 1.2 | 1.6 | 5.0 | 1.2 | 0.9 | 1.8 | 0.5 | 0.9 | 0.4 | 0.5 | 0.5 |
| Fg07295 | 1.0 | 1.3 | 2.4 | 3.0 | 7.5 | 0.8 | 0.9 | 0.6 | 0.5 | 0.7 | 0.5 | 1.2 | 1.1 |
| Fg09903 | 1.0 | 1.0 | 1.3 | 1.5 | 2.4 | 0.8 | 0.7 | 0.3 | 0.4 | 0.5 | 1.0 | 1.1 | 1.7 |
| Fg09492 | 1.0 | 1.0 | 0.8 | 0.8 | 1.1 | 1.1 | 0.7 | 0.4 | 0.4 | 0.8 | 1.7 | 0.5 | 0.6 |
| Fg06957 | 0.8 | 0.9 | 1.0 | 1.2 | 2.3 | 1.4 | 1.5 | 1.2 | 1.0 | 1.0 | 0.5 | 0.7 | 0.7 |
| Fg07344 | 1.0 | 1.0 | 0.8 | 0.8 | 0.5 | 1.2 | 0.5 | 0.3 | 0.4 | 0.4 | 1.4 | 4.8 | 2.1 |
| Fg05734 | 0.9 | 0.9 | 1.0 | 1.1 | 1.0 | 2.0 | 1.2 | 1.3 | 1.1 | 2.4 | 2.4 | 2.1 | 1.3 |
| Fg10381 | 1.2 | 1.0 | 1.0 | 0.9 | 1.0 | 1.1 | 1.0 | 0.9 | 0.7 | 0.7 | 1.0 | 2.2 | 1.8 |
| Fg00408 | 1.0 | 1.1 | 1.0 | 1.2 | 1.1 | 1.0 | 1.1 | 1.1 | 0.6 | 0.9 | 1.0 | 1.1 | 0.8 |
| Fg05484 | 0.9 | 1.0 | 0.8 | 0.9 | 1.0 | 1.8 | 1.9 | 2.1 | 1.2 | 1.3 | 2.0 | 3.0 | 3.2 |
| Fg06326 | 1.1 | 1.0 | 1.0 | 1.2 | 1.1 | 1.3 | 1.4 | 1.3 | 0.9 | 1.0 | 0.4 | 0.3 | 0.3 |
| Fg02838 | 0.8 | 0.8 | 0.7 | 0.6 | 0.5 | 1.0 | 1.0 | 1.3 | 1.3 | 1.3 | 1.0 | 1.0 | 0.9 |
| Fg10037 | 1.4 | 1.2 | 1.3 | 1.4 | 2.4 | 2.4 | 1.3 | 1.4 | 1.3 | 1.0 | 1.2 | 1.7 | 1.5 |
| Fg05135 | 0.9 | 0.9 | 0.9 | 0.9 | 0.8 | 1.4 | 1.0 | 3.8 | 4.3 | 1.1 | 0.9 | 1.0 | 1.0 |
| Fg05775 | 1.1 | 1.7 | 2.5 | 3.2 | 8.4 | 1.6 | 1.8 | 1.7 | 1.5 | 1.1 | 0.4 | 0.3 | 0.4 |
| Fg06832 | 1.0 | 1.0 | 1.0 | 1.0 | 1.3 | 0.9 | 0.9 | 0.9 | 0.9 | 0.7 | 0.3 | 0.7 | 1.0 |
| Fg05586 | 1.1 | 1.1 | 1.4 | 1.8 | 2.2 | 1.3 | 1.3 | 0.9 | 0.9 | 0.5 | 0.1 | 0.3 | 0.4 |
| Fg09408 | 1.0 | 0.9 | 0.8 | 0.8 | 0.7 | 0.8 | 0.6 | 0.5 | 0.6 | 0.8 | 2.7 | 8.7 | 1.8 |
| Fg01271 | 0.8 | 0.8 | 0.9 | 0.8 | 0.7 | 1.0 | 0.7 | 0.5 | 0.5 | 0.8 | 4.2 | 4.4 | 1.4 |
| Fg05306 | 1.0 | 1.1 | 1.5 | 1.6 | 2.4 | 1.4 | 1.2 | 1.7 | 1.3 | 1.1 | 0.4 | 0.9 | 1.2 |
| Fg10228 | 1.1 | 1.7 | 2.5 | 3.2 | 6.0 | 1.6 | 1.8 | 1.7 | 1.5 | 1.1 | 0.4 | 0.3 | 0.4 |
| Fg07520 | 1.1 | 0.9 | 1.0 | 0.8 | 1.1 | 0.9 | 1.4 | 1.1 | 1.2 | 1.6 | 0.6 | 0.6 | 1.0 |
| Fg12887 | 0.9 | 1.1 | 1.0 | 1.0 | 0.9 | 0.1 | 0.2 | 0.1 | 0.1 | 0.2 | 0.3 | 0.8 | 1.8 |
| Fg09513 | 1.1 | 1.0 | 1.0 | 1.0 | 1.0 | 1.2 | 1.0 | 0.7 | 0.7 | 0.5 | 1.2 | 1.2 | 0.9 |
| Fg09150 | 0.8 | 0.8 | 0.6 | 0.6 | 0.5 | 1.5 | 1.8 | 2.1 | 1.4 | 1.2 | 0.7 | 0.7 | 0.6 |
| Fg12132 | 0.8 | 0.8 | 0.7 | 0.8 | 0.7 | 3.5 | 20.6 | 24.3 | 3.9 | 1.5 | 1.0 | 1.0 | 1.0 |
| Fg04770 | 1.1 | 0.9 | 0.8 | 0.8 | 0.7 | 1.6 | 3.3 | 5.0 | 1.6 | 0.8 | 1.0 | 1.1 | 1.0 |
| Fg11614 | 0.9 | 0.8 | 0.7 | 0.6 | 0.6 | 1.4 | 1.6 | 0.9 | 0.7 | 0.7 | 0.4 | 0.3 | 0.4 |
| Fg07742 | 1.3 | 0.9 | 0.9 | 0.7 | 0.7 | 5.0 | 3.7 | 1.7 | 0.8 | 1.6 | 0.5 | 0.5 | 0.5 |
| Fg07745 | 0.9 | 0.7 | 0.6 | 0.6 | 0.5 | 2.6 | 6.9 | 16.7 | 5.0 | 1.6 | 1.0 | 0.9 | 0.8 |
| Fg02153 | 1.0 | 1.1 | 0.8 | 0.8 | 0.9 | 1.2 | 1.4 | 2.6 | 2.3 | 1.4 | 1.0 | 1.0 | 0.9 |
| Fg13509 | 1.1 | 0.9 | 0.8 | 0.9 | 0.7 | 11.4 | 11.1 | 13.0 | 7.1 | 3.1 | 1.1 | 1.1 | 1.0 |
| Fg10591 | 1.0 | 0.8 | 0.7 | 0.7 | 0.7 | 0.9 | 0.8 | 0.8 | 0.6 | 0.5 | 1.1 | 1.1 | 1.1 |
| Fg00362 | 1.1 | 1.1 | 0.9 | 1.0 | 1.0 | 1.0 | 1.1 | 1.0 | 1.0 | 1.1 | 1.4 | 1.1 | 1.1 |
| Fg06420 | 1.0 | 1.0 | 0.9 | 1.0 | 1.0 | 0.8 | 1.0 | 0.6 | 0.4 | 0.6 | 0.2 | 0.3 | 0.8 |
| Fg00792 | 1.1 | 0.9 | 0.8 | 0.9 | 0.9 | 0.6 | 1.0 | 0.9 | 0.6 | 0.5 | 0.9 | 0.6 | 1.8 |
| Fg01559 | 0.6 | 0.7 | 0.6 | 0.6 | 0.5 | 1.2 | 3.6 | 7.6 | 18.9 | 14.0 | 1.1 | 1.0 | 0.8 |
| Fg01641 | 0.8 | 0.6 | 0.7 | 0.6 | 0.6 | 1.0 | 1.3 | 0.6 | 0.6 | 0.9 | 0.2 | 0.1 | 0.1 |
| Fg10196 | 0.8 | 0.7 | 0.7 | 0.7 | 0.7 | 1.5 | 1.2 | 1.3 | 1.4 | 1.8 | 1.3 | 2.1 | 1.1 |
| Fg03499 | 1.0 | 0.8 | 0.8 | 0.8 | 0.6 | 1.0 | 1.0 | 1.2 | 3.8 | 76.8 | 1.1 | 1.3 | 1.1 |
| Fg11394 | 0.8 | 0.8 | 0.6 | 0.7 | 0.6 | 1.0 | 0.9 | 0.8 | 0.9 | 0.9 | 1.1 | 1.0 | 0.9 |
| Fg07381 | 1.2 | 1.2 | 1.4 | 1.4 | 1.7 | 1.8 | 1.5 | 1.4 | 0.8 | 1.6 | 0.4 | 1.3 | 1.9 |
| Fg01963 | 1.1 | 1.2 | 1.1 | 1.2 | 1.9 | 1.1 | 0.6 | 0.5 | 0.5 | 0.5 | 0.8 | 0.9 | 0.9 |
| Fg04416 | 1.0 | 1.1 | 0.9 | 0.8 | 0.8 | 1.1 | 1.0 | 1.4 | 1.2 | 0.8 | 0.2 | 0.3 | 0.3 |
| Fg13318 | 1.0 | 1.1 | 0.8 | 0.8 | 0.8 | 1.8 | 1.2 | 1.8 | 1.7 | 3.2 | 3.2 | 2.4 | 0.9 |
| Fg05519 | 1.0 | 0.9 | 0.8 | 1.0 | 0.9 | 2.3 | 1.9 | 2.4 | 2.0 | 1.5 | 0.7 | 0.6 | 0.5 |
| Fg08133 | 1.0 | 1.0 | 0.9 | 1.0 | 1.3 | 1.2 | 1.1 | 1.2 | 0.8 | 1.1 | 0.5 | 0.7 | 0.4 |
| Fg06089 | 1.0 | 1.3 | 1.2 | 1.7 | 2.7 | 1.4 | 1.7 | 2.6 | 2.2 | 2.3 | 1.1 | 0.5 | 0.4 |
| Fg06502 | 1.0 | 0.8 | 0.8 | 1.0 | 1.2 | 1.6 | 1.6 | 0.7 | 0.6 | 1.5 | 8.1 | 0.9 | 0.6 |
