# Supplementary material for: Functional Analysis of the Kinome of the Wheat Scab Fungus Fusarium graminearum
Source: PLoS Pathog. 2011 Dec 22;7(12):e1002460. doi: 10.1371/journal.ppat.1002460 (PMC3245316; doi:10.1371/journal.ppat.1002460)
Supplement: Table S4 — The Pearson correlation efficient between major phenotypes. (DOC) [file ppat.1002460.s008.doc]

**Table S4. The Pearson Correlation Efficient between Major Phenotypes**

|  | **ST^a^** | **GT** | **CM** | **CON** | **HYP** | **DON** | **SR** | **VIR** | **GR** |
| --- | --- | --- | --- | --- | --- | --- | --- | --- | --- |
| ST | 1 | 0.187 | 0.398^*^ **^b^** | 0.173 | 0.150 | 0.507^*^ | 0.591^*^ | 0.620^*^ | 0.639^*^ |
| GT | - | 1 | 0.273^*^ | 0.450^*^ | 0.103 | 0.166 | 0.202 | 0.161 | 0.105 |
| CM | - | - | 1 | 0.351^*^ | 0.200 | 0.472^*^ | 0.472^*^ | 0.444^*^ | 0.487^*^ |
| CON | - | - | - | 1 | 0.064 | 0.266^*^ | 0.266^*^ | 0.303^*^ | 0.271^*^ |
| HYP | - | - | - | - | 1 | 0.103 | 0.201 | 0.127 | 0.109 |
| DON | - | - | - | - | - | 1 | 0.506^*^ | 0.639^*^ | 0.576^*^ |
| SR | - | - | - | - | - | - | 1 | 0.695^*^ | 0.727^*^ |
| VIR | - | - | - | - |  | - | - | 1 | 0.736^*^ |
| GR | - | - | - | - | - | - | - | - | 1 |

**^a^**ST: Responses to hyperosmotic stress; GT: Conidium germination and germ tube growth; CM: Conidium morphology; CON: Conidiation; HYP: Hyphal tip growth and branching; DON: DON production; SR: sexual reproduction; VIR: virulence; GR: growth rate.

**^b^** The Pearson correlation coefficient was calculated with SPSS. Symbols* indicate significant correlation at the 0.01 confidence level.
