# Supplementary material for: Functional Analysis of the Kinome of the Wheat Scab Fungus Fusarium graminearum
Source: PLoS Pathog. 2011 Dec 22;7(12):e1002460. doi: 10.1371/journal.ppat.1002460 (PMC3245316; doi:10.1371/journal.ppat.1002460)
Supplement: Table S5 — Mutants with defects in germ tube (GT) growth. (DOC) [file ppat.1002460.s009.doc]

**Table S5. Mutants with Defects in Germ Tube (GT) Growth**

| **Defects*** | **Mutants** |
| --- | --- |
| **Short germ tubes, reduced GT branching** | Fg01641 (multiple GTs)  Fg09897 (multiple GTs)  Fg10037 (Bud32)  Fg07329 (Gsk3)  Fg10228 (Swe1)  Fg04053 (Prp4)  Fg07251 (CpkA) |
| **Abnormal GT morphology** | Fg04382 (Fpk1) GT growth normal but less branching  Fg01312 (wavy GTs)  Fg07251 (CpkA)  Fg09897 (intercalary and apical swelling)  Fg07329 (wavy GTs) |

* Germ tube growth and branching were observed after germinating in CM at 25^o^C for 12 h.
