# Supplementary material for: Functional Analysis of the Kinome of the Wheat Scab Fungus Fusarium graminearum
Source: PLoS Pathog. 2011 Dec 22;7(12):e1002460. doi: 10.1371/journal.ppat.1002460 (PMC3245316; doi:10.1371/journal.ppat.1002460)
Supplement: Table S6 — Mutants with altered responses to stresses. (DOC) [file ppat.1002460.s010.doc]

**Table S6. Mutants with altered responses to stresses**

| **Stresses** | **Mutants with 40% changes ^a^** | |
| --- | --- | --- |
|  | Increased sensitivity | Increased tolerance |
| **0.7 M NaCl** | Fg06939  Fg04382  Fg09612 (ng) **^b^**  Fg08691 (ng)  Fg00408 (ng)  Fg09274 | Fg01641  Fg09897  Fg04947  Fg08906  Fg04484 |
| **0.05% H_2_O_2_** | Fg00472  Fg04382  Fg05418  Fg13318  Fg07329 **^c^**  Fg10037 | Fg05734  Fg08701  Fg00469 |

**^a^** Vegetative growth was reduced or increased more than 40% than the wild type.

**^b^** ng, no visible growth.

**^c^** The Fg07329 and Fg10037 mutants had no visible growth in the presence of 0.05% H_2_O_2_.
