# Supplementary material for: Functional Analysis of the Kinome of the Wheat Scab Fungus Fusarium graminearum
Source: PLoS Pathog. 2011 Dec 22;7(12):e1002460. doi: 10.1371/journal.ppat.1002460 (PMC3245316; doi:10.1371/journal.ppat.1002460)
Supplement: Table S7 — Kinase mutants with 30% reduction in DON production. (DOC) [file ppat.1002460.s011.doc]

**Table S7. Kinase mutants with 30% reduction in DON production**

| **FGSG** | **DON (%) ^a^** |
| --- | --- |
| Fg04947 | 1.5 * |
| Fg06939 | 29.3 * |
| Fg01312 | 33.5 * |
| Fg07812 | 34.0 |
| Fg06970 | 37.6 |
| Fg00472 | 38.2 * |
| Fg12149 | 43.8 |
| Fg06420 | 47.8 |
| Fg05586 | 49.7 |
| Fg04770 | 49.9 * |
| Fg01347 | 50.2 |
| Fg02488 | 50.9 |
| Fg10095 | 51.9 * |
| Fg01559 | 52.7 |
| Fg08729 | 53.2 |
| Fg06793 | 53.7 * |
| Fg00792 | 55.8 |
| Fg05519 | 56.5 |
| Fg01506 | 56.6 |
| Fg03132 | 58.3 |
| Fg06878 | 58.8 * |
| Fg00469 | 59.6 |
| Fg00786 | 60.5 * |
| Fg13509 | 62.6 |
| Fg07381 | 62.7 |
| Fg10591 | 63.0 |
| Fg08701 | 63.3 |
| Fg08631 | 64.2 |
| Fg11614 | 65.4 |
| Fg04416 | 66.1 |
| Fg00433 | 66.6 |
| Fg02153 | 67.4 |
| Fg05734 | 69.6 |

**^a^** Percentage of DON production in the mutants in comparison with the wild tye. DON was assayed with infested kernels only for mutants with a disease index larger than 1.5. Mutants with a disease index less than 5 are marked with symbol *.
