# Supplementary material for: Functional Analysis of the Kinome of the Wheat Scab Fungus Fusarium graminearum
Source: PLoS Pathog. 2011 Dec 22;7(12):e1002460. doi: 10.1371/journal.ppat.1002460 (PMC3245316; doi:10.1371/journal.ppat.1002460)
Supplement: Table S8 — Mutant phenotypes of 28 F graminearum PK genes with no distinct orthologs in S. cerevisiae. (DOC) [file ppat.1002460.s012.doc]

**Table S8. Mutant phenotypes of 28 *F graminearum* PK genes with no distinct orthologs in *S. cerevisiae*.**

| **Gene** | **Ortholog in *S. pombe*** | **Mutant Phenotype** |
| --- | --- | --- |
| Fg00132 |  | nd **^*^** |
| Fg00362 |  | Defects in growth, conidiation, pathogenicity, and sexual reproduction |
| Fg00792 |  | Reduced in DON production |
| Fg01058 |  | Defective in ascospore morphology and release |
| Fg01559 |  | Reduced in DON production |
| Fg02153 |  | nd |
| Fg02488 |  | Reduced in DON production |
| Fg02838 |  | nd |
| Fg03146 |  | Slightly reduced in conidiation and virulence |
| Fg03499 |  | nd |
| Fg04053 | *prp4* | Several growth defects. Nonpathogenic |
| Fg04416 |  | nd |
| Fg04770 |  | Reduced in virulence and DON production |
| Fg05406 | *ppk23* | Reduced in conidiation |
| Fg05549 |  | nd |
| Fg06420 |  | Reduced in DON production |
| Fg07344 | *sid1* | Defects in growth, conidiation, pathogenicity, and sexual reproduction |
| Fg07742 |  | nd |
| Fg07745 |  | nd |
| Fg07812 |  | nd |
| Fg09150 |  | Approximately 80% reduction in conidiation |
| Fg10095 | *pom1* | Defective in growth and conidiation, reduced virulence and blocked in ascospore release |
| Fg10591 |  | nd |
| Fg11614 |  | nd |
| Fg12132 |  | nd |
| Fg12887 |  | nd |
| Fg13509 |  | nd |
| Fg13944 |  | nd |

**^*^**nd, no detectable phenotype.
